# A population genomics analysis of the Aotearoa New Zealand endemic rewarewa tree (*Knightia excelsa*)

**DOI:** 10.1038/s44185-024-00038-6

**Published:** 2024-03-20

**Authors:** Ann M. McCartney, Emily Koot, Jessica M. Prebble, Rubina Jibran, Caroline Mitchell, Ana Podolyan, Alexander J. Fergus, Elise Arnst, Katie E. Herron, Gary Houliston, Thomas R. Buckley, David Chagné

**Affiliations:** 1 Manaaki Whenua - Landcare Research, 231 Morrin Road, Saint Johns, Auckland, 1072 New Zealand; 2 Genomics Aotearoa, Aotearoa, New Zealand; 3 Genomics Institute, University of California, Santa Cruz, CA 95060 USA; 4 The New Zealand Institute for Plant and Food Research Limited (Plant & Food Research), Batchelar Road, Fitzherbert, Palmerston North, 4474 New Zealand; 5 Manaaki Whenua - Landcare Research, 54 Gerald Street, Lincoln, 7608 New Zealand; 6 Plant & Food Research, 120 Mt Albert Road, Sandringham, Auckland, 1025 New Zealand; 7 School of Biology and Environmental Science, University College, Dublin, Ireland

**Keywords:** Natural variation in plants, Plant genetics, Ecological genetics

## Abstract

Rewarewa (*Knightia excelsa*, Proteaceae) is a tree species endemic to Aotearoa New Zealand, with a natural distribution spanning Te Ika-a-Māui (North Island) and the top of Te Waipounamu (South Island). We used the pseudo-chromosome genome assembly of rewarewa as a reference and whole genome pooled sequencing from 35 populations sampled across Aotearoa New Zealand, including trees growing on Māori-owned land, to identify 1,443,255 single nucleotide polymorphisms (SNPs). Four genetic clusters located in the northern North Island (NNI), eastern North Island (NIE), western and southern North Island (NIWS), and the South Island (SI) were identified. Gene flow was revealed between the SI and NIE genetic clusters, plus bottleneck and contraction events within the genetic clusters since the mid-late Pleistocene, with divergence between North and South Island clusters estimated to have occurred ~115,000–230,000 years ago. Genotype environment analysis (GEA) was used to identify loci and genes linked with altitude, soil pH, soil carbon, slope, soil size, annual mean temperature, mean diurnal range, isothermality, annual precipitation, and precipitation seasonality. The location of the SNPs associated with these environmental variables was compared with the position of 52,192 gene-coding sequences that were predicted in the rewarewa genome using RNA sequencing. This new understanding of the genetic variation present in rewarewa and insights into the genetic control of adaptive traits will inform efforts to incorporate the species in restoration plantings and for marketing rewarewa honey based on provenance.

## Introduction

The rewarewa tree (New Zealand honeysuckle, *Knightia excelsa* R.Br., Proteaceae) is endemic to Aotearoa New Zealand, where its range extends throughout Te Ika-a-Māui (North Island) and into the top of Te Waipounamu (South Island). The te reo Māori word “rewa” refers to the flower of the rewarewa tree; this was then extended to rewarewa to refer to the whole tree. It is a monotypic endemic genus^1^, that diverged during the Paleogene (~60 million years ago); its nearest relatives remain unclear^2^. Although previously considered con-generic with two New Caledonian species, more recent molecular phylogenies clearly show they are not each other’s nearest relatives^2,3^, and the New Caledonian species have now been placed in the genus *Eucarpha*. The latest classification, therefore, places *K. excelsa* R.Br. as *incertae sedis* within the Roupaleae tribe of the Grevilleoideae subfamily of Proteaceae^3^.

**Fig. 1:**
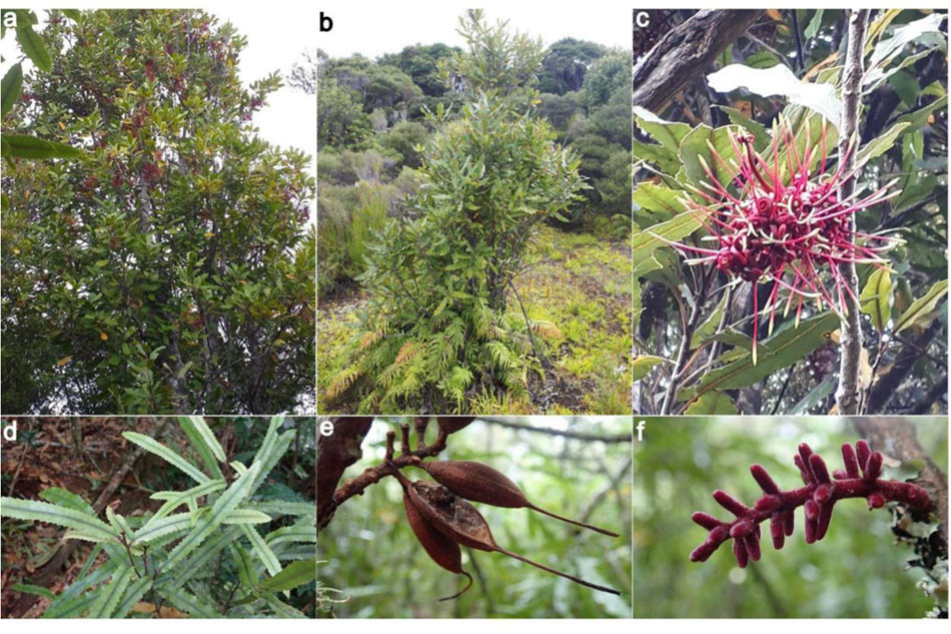
Photo plate illustrating typical features of rewarewa. **a** Adult tree in flower, **b** tree that the genome assembly of McCartney et al. was based on, **c** inflorescence showing the prominent yellow-tipped styles. **d** juvenile leaves - generally longer and thinner than adult leaves. **e** Dry lignified follicles with persistent styles after the papery seeds have already dispersed, and **f** inflorescence in bud. Photo credits: **a**, **d**, **e**, and **f**, by A. J. Fergus; **b** and **c** by J. M. Prebble.

Rewarewa are a common tree at coastal and inland sites, and are particularly evident at forest regeneration sites: they form dense stands when colonizing, e.g., after fire events^4^, then reduce to a more minor part of the canopy as the forest becomes more established. A rapidly growing tree, rewarewa can reach a height of 30 meters at full maturity. Noticeable characteristics include their conical shape, thick leathery toothed leaves, and conspicuous inflorescences made up of many red or red-pink flowers with prominent styles. Their papery seeds are wind dispersed^5^ and contained in dry wooden follicles (Fig. 1).

Nectar is produced at the base of rewarewa flowers, and traditionally, this nectar was collected by Māori as a sweetening agent or sipped to heal sore throats (manawahoney.co.nz)^6^, hence the European common name of ‘New Zealand honeysuckle’. Rewarewa’s nectar-producing capabilities make it attractive to bees but also result in the tree being commonly frequented (and pollinated) by endemic nectar-feeding birds such as the tūī (*Prosthemadera novaeseelandiae*), korimako (bellbird; *Anthornis melanura*), tauhao (silvereye/waxeye; *Zosterops lateralis*)^7^ and even one of Aotearoa’s very few endemic mammals, pekapeka-tou-poto (short-tailed bats; *Mystacina tuberculata*^8^). The nectar produced has an amber color and a smooth malty flavor^7^. Research suggests that rewarewa honey has a range of properties from antibacterial to antioxidant and anti-inflammatory^9^, and further research is ongoing in this area.

To aid research in this species, the first pseudo-chromosomal genome of rewarewa was recently assembled^10^. This is a first, and significant step toward generating more genomic resources for rewarewa as prior to this; the most closely related published genome was for that of *Macadamia integrifolia*^11^ (Proteaceae).

In this study, we used the published rewarewa genome assembly as a reference to identify single nucleotide polymorphisms (SNPs) from 35 populations sampled across the natural range of rewarewa, using 15 pooled individual samples per site. An understanding of the genetic variation present in this species and how the variation is partitioned will aid in determining how regional variation affects the species adaptation to the environment. Transcriptome analysis was used for predicting gene-coding regions in the rewarewa genome, and genotype-environment analysis (GEA) was used to identify loci and genes linked with environmental conditions.

## Results

### Variant detection

**Fig. 2:**
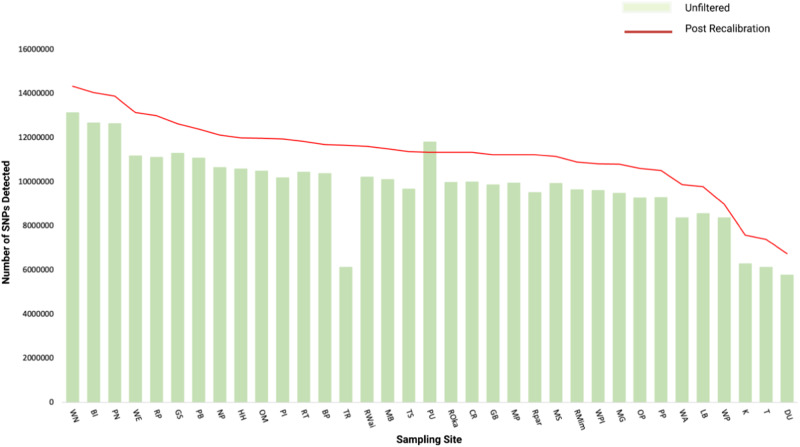
Total frequency of SNPs called across 35 rewarewa populations before and after filters were applied.

Through an initial round of variant detection using GATK (v4.1.8.1)^12^ pooled sequencing aware protocol, a total of 343,622,891 SNPs were called across the 35 populations. The average number of SNPs called across the 35 populations was 9,817,797, with the six highest SNP calls belonging to populations sampled from the most northerly region of the North Island (Fig. 2). SNPs were then filtered, recalibrated, and recalled. As a result of this recalibration, the total number of SNPs across the 35 populations was 11,248,183 on average, increasing the total number of SNPs by 1,430,386 per population. However, for the PU population, the recalibration actually resulted in a decrease of SNPs called by 476,555.

### Genetic diversity and population structure

**Fig. 3:**
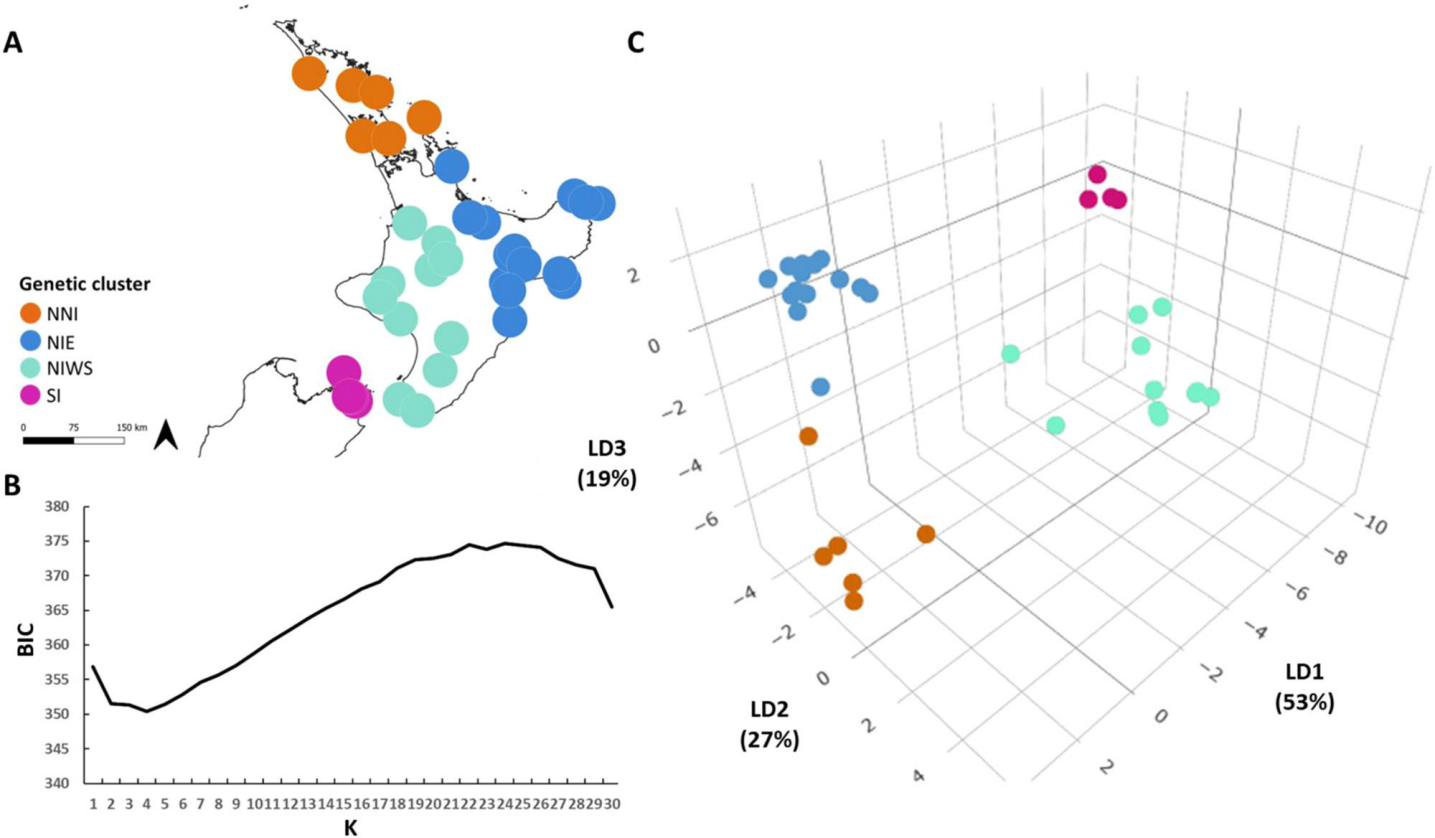
Population structure of Aotearoa New Zealand endemic tree species *Knightia excelsa* based on whole genome pooled sequencing. **A** Sampling locations of clusters colored by genetic clusters identified by K-means clustering analysis. Points are purposefully enlarged to address the sensitivity associated with indigenous intellectual property. **B** Broken elbow plot of Bayesian Information Criterion (BIC) scores from K-means clustering analysis used to determine optimum number of clusters within the dataset. **C** Discriminant Analysis of Principal Components (DAPC) (*K* = 4) for *K. excelsa*. Linear discriminants (LD) 1, 2 and 3 accounting for 53%, 27%, and 19% of variation within the dataset, respectively. Points are colored by the genetic cluster identified by *K* means clustering analysis: Northern North Island (NNI), eastern North Island (NIE), western and Southern North Island (NIWS), and South Island (SI).

**Fig. 4:**
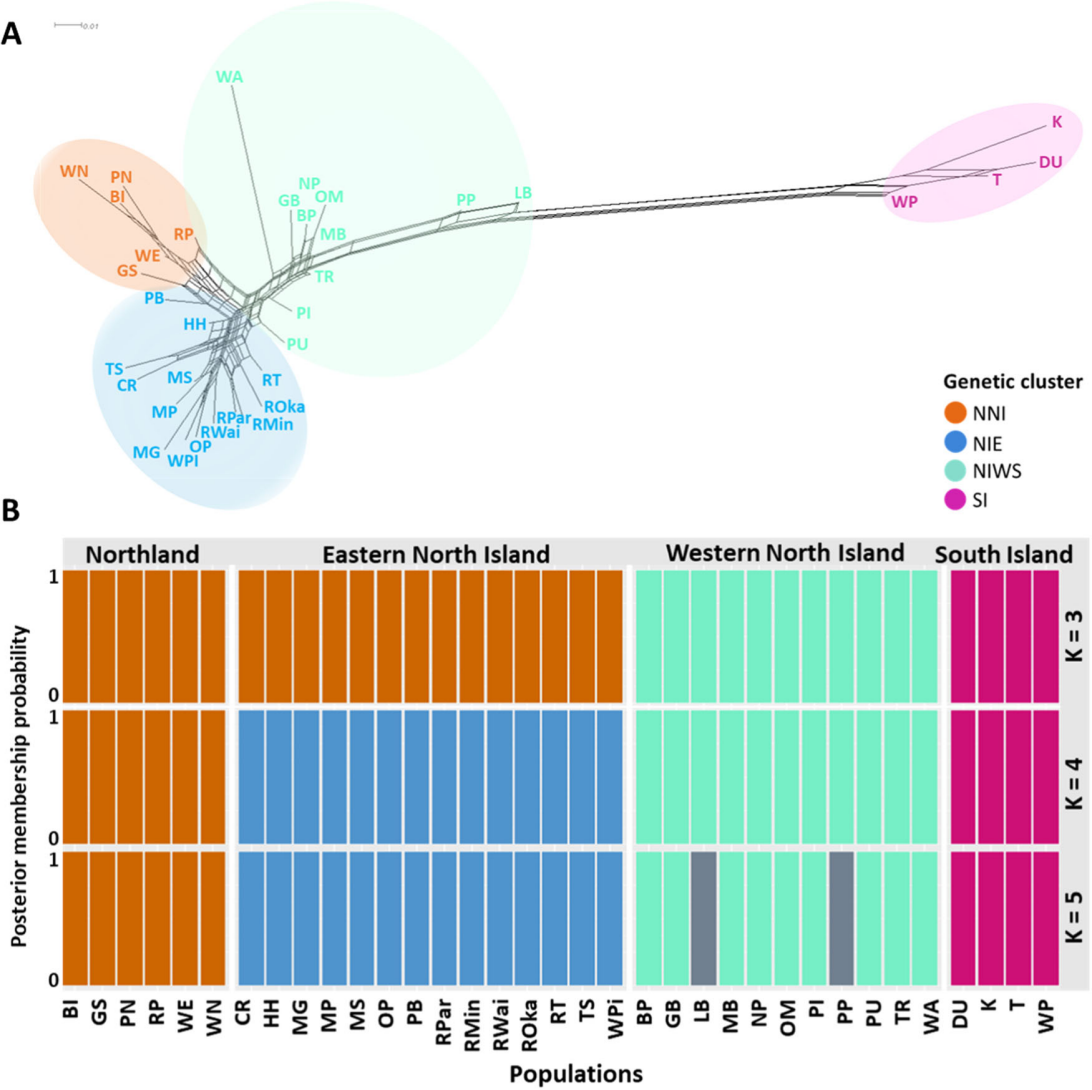
SplitsTree neighbor-joining network and posterior-membership probability. **A** SplitsTree neighbor-joining network of *Knightia excelsa* indicating population structure and differentiation based on pairwise *Fst* genetic distances. **B** Stacked bar plots of posterior-membership probability for each population for three K values (3–5). SplitsTree tips and bar plot bars are colored by genetic clusters identified by the K-means clustering analysis: Northern North Island (NNI), eastern North Island populations (NIE), western and southern North Island (NIWS) and South Island (SI).

Following filtering, 1,443,255 SNPs remained for the population genomic analyses. The Adegenet (v2.1.1)^13^ k-means clustering analysis determined *K* = 4 to be the optimum number of clusters within the dataset (out of 30), based on having the lowest Bayesian Information Criterion (BIC) score (BIC = 350.41) (Fig. 3B). This optimal *K* value was applied to the discriminant analysis of principal components (DAPC)^14^, in addition to an optimized number of principal components (PC) (PC = 5), to reveal that the dataset segregates into four genetically and geographically distinct genetic clusters (Fig. 3A, C). Three Linear Dimensions (LD) were retained in the analysis (LD1, LD2, LD3), and these explained 53%, 27%, and 19% of the data, respectively. The four genetic clusters identified by this analysis consist of: (1) northern North Island populations (NNI), (2) eastern North Island populations (NIE), (3) western and southern North Island populations (NIWS), and (4) South Island populations (SI). Two additional *K* values were explored—*K* = 3 and *K* = 5 (Fig. 4B). When *K* = 3 was applied to the DAPC analysis, NNI and NIE clusters formed a single cluster. When *K* = 5 was applied, an additional cluster was revealed within the NIWS genetic cluster, consisting of two populations from the Wellington and Wairarapa regions (southern North Island)—LB and PP.

Pairwise *Fst* genetic distances were estimated between all populations using PoolFstat (v1.0.0)^15^; these were then averaged by genetic cluster based on results of the k-means clustering and DAPC analyses. Pairwise *Fst* distances between all populations can be found in Supplementary Table 1. Global *Fst* across all populations was 0.128 (SD = 0.09) (sd = 0.05), whilst mean pairwise *Fst* differences between North and South Island populations was 0.309. The lowest pairwise differences were between NNI and NIE (mean pairwise *Fst* = 0.086, SD = 0.02), whilst the highest differences were between NNI and SI (mean pairwise *Fst* = 0.334, SD = 0.03) (Table 1). Distances were also relatively high between the other two North Island genetic clusters and the South Island genetic cluster (NIE & SI = 0.327, NIWS & SI = 0.27). A SplitsTree (v4.14.8)^16^ neighbor-joining network of the pairwise *Fst* distances supports these findings, with the South Island populations being genetically distinct from all North Island populations (Fig. 4A). Within the North Island samples, the North Island populations cluster into the three groupings identified in the DAPC analysis. An Isolation By Distance (IBD)^17^ analysis revealed there to be no significant relationship between geographic and pairwise *Fst* genetic distances (*r* = 0.078, *p* value = 0.113).

**Table 1:** Mean pairwise fixation index (*Fst*) as a measure of population differentiation between genetic clusters

	NNI	NIE	NIWS	SI
NNI	0.049 (0.01)			
NIE	0.086 (0.02)	0.054 (0.02)		
NIWS	0.109 (0.04)	0.095 (0.03)	0.061 (0.04)	
SI	0.334 (0.03)	0.327 (0.04)	0.272 (0.05)	0.066 (0.03)

Pairwise *Fst* distances between all populations can be found in Supplementary Table 1. Standard deviations are in brackets. Northern North Island (NNI), eastern North Island (NIE), western and southern North Island (NIWS) and South Island (SI).

Summary and neutrality statistics were calculated for all populations using NPStats v1^18^, and are presented here as weighted medians averaged by genetic cluster (Table 2). Weighted medians for all populations can be found in Supplementary Table 2. The average number of segregating sites (S) ranged from 188.27 (SD = 13.7) (NIWS) to 235.17 (SD = 13.12) (NNI). Average Watterson’s *θ* ranged from 0.0053 (SD = 0.0004) (NIWS) to 0.0066 (sd = 0.0003) (NNI). Average nucleotide diversity (*π*) ranged from 0.0042 (SD = 0.0004) (NIWS) to 0.0052 (SD = 0.0001) (NNI), and average Tajima’s D ranged from -0.819 (SD = 0.087) (NNI and NIWS) to −0.722 (SD = 0.08) (NIE).

**Table 2:** NPStats summary and neutrality statistics weighted medians averaged by genetic cluster for *Knightia excelsa* populations

Region	S	Watterson’s θ	*π*	Tajima D
NNI	235.17 (13.12)	0.0066 (0.0003)	0.0052 (0.0001)	−0.819 (0.09)
NIE	193.50 (11.32)	0.0054 (0.0003)	0.0044 (0.0002)	−0.722 (0.08)
NIWS	188.27 (13.7)	0.0053 (0.0004)	0.0042 (0.0004)	−0.819 (0.12)
SI	200.97 (22.76)	0.0056 (0.0006)	0.0045 (0.0003)	−0.777 (0.09)

*S* segregating sites, *π* nucleotide diversity, *SD* standard deviation. Standard deviations are in brackets. Summary statistics for each population can be found in Supplementary Table 2.

**Fig. 5:**
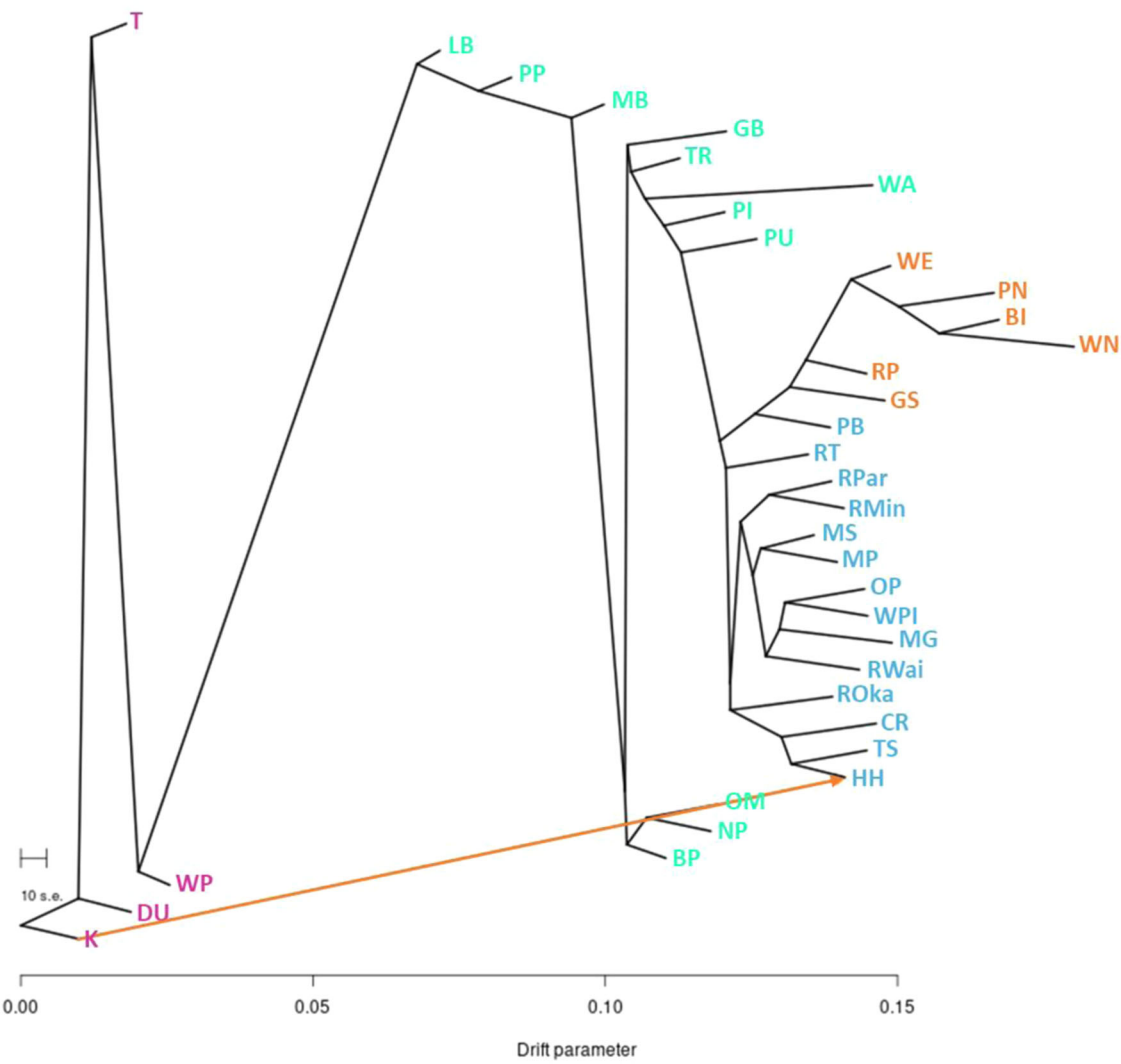
TreeMix analysis of migration events between *Knightia excelsa* clusters. Phylogenetic representation of TreeMix outputs with the optimum number of migration events displayed (*M* = 1). Tree tips are colored by the genetic cluster identified by the K-means clustering analysis: Northern North Island (NNI), eastern North Island populations (NIE), western and southern North Island (NIWS), and South Island (SI). The scale bar shows 10x the average standard error (s.e.), and drift parameter is shown on the *x* axis.

Thirty migration events were explored by the TreeMix (v1.13)^19^ analysis, with one migration event deemed best to explain the observed variance within the dataset according to OptM (v0.1.6)^20^, with a delta m score of 17.62 (Fig. 5). The best migration event was from population K in the South Island, to population HH in eastern North Island. The phylogenetic tree produced by the analysis has a similar topology to the Splitstree network, with the South Island populations being clearly distinct from the North Island populations. The clustering of North Island populations matched that of the genetic clusters identified by the K-means clustering and DAPC analyses. The second highest delta m score was for *M* = 3 (delta *m* = 2.8), with two additional migration edges occurring from population GB to RP, and populations RP/WN to PI.

### Demographic analysis

Four demographic hypotheses were explored for each genetic cluster using fastsimcoal2 (v2.6.0.3)^21^, with results suggesting that three genetic clusters (NNI, NIWS, NIE) have historically undergone contraction events, and one has undergone a bottleneck (SI) (Supplementary Tables 3 and 4). Each model was run 100 times, before the best model (chosen based on AIC) was run an additional 100 times. Here, we present the results of the best run from those final 100 runs for each genetic cluster. NNI is estimated to have begun contracting ~74,060 generations ago, with effective population size (Ne) reducing by 39%; NIWS began contracting ~51,302 generations ago, with Ne reducing by 32.5%; and NIE began contracting 73,965 generations ago, with a reduction in Ne by 36.5%. SI is predicted to have undergone a bottleneck event approximately 137,986 generations ago, with this bottleneck ending approximately 58,034 generations ago. During the bottleneck, Ne was reduced by 75.5%; however, following the end of the bottleneck, Ne is estimated to have increased 400%, and surpassed ancestral Ne by 23.5%. For the three genetic clusters that have undergone population contractions, results for bottleneck and contraction models were very similar, and in some instances, the bottleneck model had lower AIC values. However, results of the bottleneck modeling indicated that the effective population size of the gene pools did not recover following the bottleneck, and was less than the effective population size during the bottleneck—effectively indicating that the population sizes have continued to contract (hence the contraction model being selected as the more suitable model). Of the four models used to explore the demographic history of divergence and gene flow between North and South Island populations, the divergence followed by isolation (D-I) model was determined to best explain the evolutionary history of these populations. It is estimated that these populations diverged ~22,748 generations ago, with the NIWS genetic cluster subsequently contracting by 6.2%, and the SI population expanding by 55.1%.

### Gene predictions

The rewarewa genome was annotated using RNA sequencing. Reads were assessed for quality and were trimmed for Illumina Universal Adaptors using trimgalore (http://www.bioinformatics.babraham.ac.uk) (v0.6.7). Although duplicated sequences were identified, this was expected to be a consequence of the RNAseq data having multiple transcripts as opposed to an artifact of the reads themselves. The remaining 130 bp paired-end Illumina reads (272,672,080 in total) were aligned to the complete pseudo-chromosomal reference assembly for rewarewa using STAR (v7.10)^22^. Alignment statistics were generated suggesting 100% of these reads passed Illumina filters and were mapped to the reference. We accurately mapped 72% of these reads using >Q20 as a cut-off, as this would result in 1/100 chance that incorrect mapping has occurred. Additionally, the statistics suggested that there was no bias in regard to the ability of strands to be mapped. We then conducted an annotation of the assembly resulting in the identification of 52,192 genes, 288,686 exons, 288,686 CDS, 232,075 introns, 56,602 start codons, 56,592 stop codons, and 56,633 transcripts. To give an indication of annotation quality, the BRAKER3 (v3)^23^ annotation output was assessed using BUSCO (v5.4.4)^24^ (Eukaryote and Eudicot databases), and the result was compared to the BUSCO result from the reference genome assembly (Table 3).

**Table 3:** BUSCO analysis results comparing BUSCO genes identified in the genome assembly for *Knightia excelsa* vs the BRAKER3 annotated genes

	Eukaryote BUSCO annotation assessment	Eukaryote BUSCO genome assembly	Eudicot BUSCO annotation assessment	Eudicot BUSCO genome assembly
Complete BUSCO	246 (96.5%)	100%	1866 (80.2%)	96.7%
Complete and single-copy BUSCO (S)	87 (34.1%)	23.9%	1315 (56.5%)	78.6%
Complete and duplicated BUSCO (D)	159 (62.4%)	76.1%	551 (23.7%)	18.1%
Fragmented BUSCO (F)	8 (3.1%)	0%	181 (7.8%)	1.1%
Missing BUSCO (M)	1 (0.4%)	0%	279 (12%)	2.2%
Total BUSCO searched	255	255	2326	2326

### Genotype environment associations

**Fig. 6:**
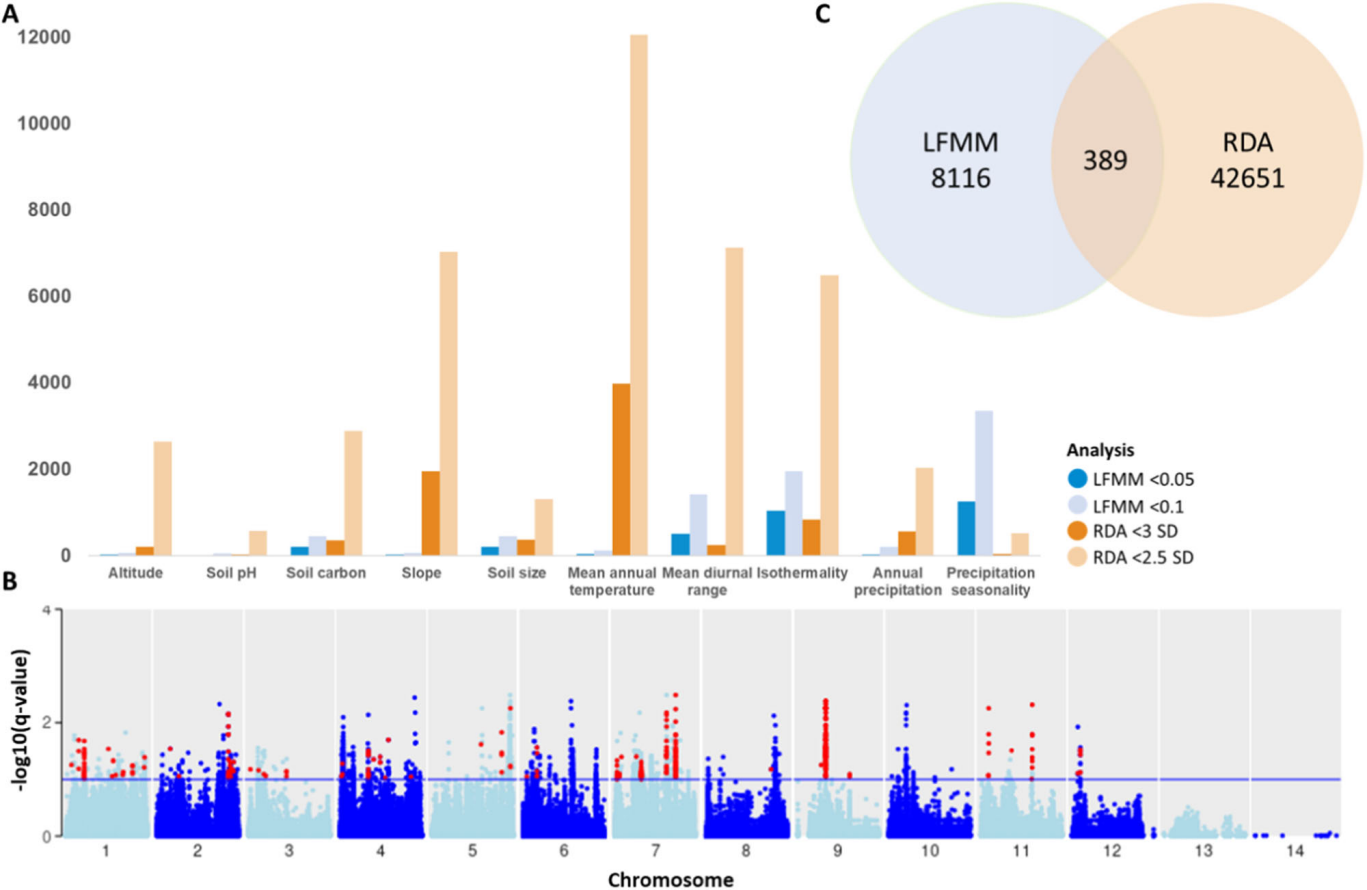
Genotype environment association results for latent factor mixed model (LFMM) and redundancy analyses (RDA) of *Knightia excelsa* populations. **A** Results for two different thresholds for both LFMM (*q* value < 0.05 and <0.1) and RDA (3 standard deviations (SD) and 2.5 SD). Specific results can be found in Supplementary Tables 5 and 6. **B** Manhattan plot of LFMM (threshold *q* value < 0.1) −log10 *q* values for mean diurnal range, points in red indicate SNPs that were also identified as significant by RDA (threshold 2.5 SD) analysis. **C** Intersect of significant SNPs identified by the LFFM (threshold q value < 0.1) and RDA (threshold 2.5 SD), which were further investigated in the study.

After checking for collinearity between the initial variables, 10 variables were deemed suitable for use in the GEA analysis: altitude, soil pH, soil carbon, slope, soil size, mean annual temperature, mean diurnal range, isothermality, annual precipitation and precipitation seasonality. The Latent Factor Mixed Model (LFMM) analysis in lfmm2^25^ identified 3346 and 8116 significant SNPs, when thresholds of *q* values < 0.05 and <0.1 were applied, respectively. In contrast, the Redundancy Analysis (RDA) identified 8544 and 42651 SNPs when thresholds of 3 SD and 2.5 SD were explored, respectively. Only 10 SNPs were identified by both analyses as being significant when the more stringent thresholds were applied (*q* value < 0.05 for LFMM and 2.5 SD for RDA), however, when the less stringent thresholds were applied (*q* value < 0.05 for LFMM and 2.5 SD for RDA), 389 SNPs were identified (Fig. 6A, C, Supplementary Table 5). These 389 SNPs were further explored. The variable to have the most SNPs associated with it was mean diurnal range (335 SNPs across 29 loci), followed by Isothermality (23 SNPs across 13 loci) and precipitation seasonality (16 SNPs across 8 loci) (Supplementary Table 6). Altitude, mean annual temperature and soil size had six (three loci), four (three loci) and three (one loci) SNPs associated with them, respectively, whilst soil pH and annual precipitation only had one associated SNP. Soil carbon and slope were found to have no associated SNPs.

Of the 389 SNPs, 51 were located close to or within predicted gene models (Supplementary Table 7). Genes linked to SNPs that were significantly associated with environmental variables, as indicated by a high redundancy analysis (RDA) loading value, are shown in Table 4, with putative functions determined from homology with *Arabidopsis thaliana*. Out of the loci identified by GEA, nine genes were detected, which include homologs of a *TRF-like* gene, a *O-fucosyltransferase*, an *E3 ubiquitin ligase*, a *cyclin-dependent kinase*, a *jacalin-related lectin* and a *SAM methyltransferase*. The SNPs associated with the mean diurnal range were primarily found in genes governing various aspects of plant growth and development, such as abiotic stress response and hormone regulation. Five of the SNPs were located in exons, including two SNPs that produced amino acid changes.

**Table 4:** Predicted genes associated with environmental variables in the rewarewa (*Knightia excelsa*) genome

Variable	Genomic region	Predicted gene model ID	*Arabidopsis* homolog	Putative function	RDA Loading Value		Effect of SNP on protein
Mean diurnal range	Intron	g51191	AT1G72650	TRF-like 6 (stress response)		0.80	n/a
Mean diurnal range	Intron	g29343	AT1G48580	Unknown function		0.76	n/a
Mean diurnal range	Intron	g5962	AT1G52630	O-fucosyltransferase		0.77	n/a
Mean diurnal range	Exon	g926	AT3G28840	Unknown function		0.87	AA change
Mean diurnal range	Exon	g35014	AT5G49350	E3 ubiquitin ligases		0.70	synonymous change
Mean diurnal range	Exon	g38661	AT5G49350	E3 ubiquitin ligases		0.71	AA change
Precipitation seasonality	Intron	g29008	AT4g23460	cyclin-dependent kinase		0.62	n/a
Precipitation seasonality	Intron	g5001	AT3G16460	JAL34 jacalin-related lectin 34		0.76	n/a
Precipitation seasonality	Intron	g5001	AT3G16460	JAL34 jacalin-related lectin 34		0.76	n/a
Precipitation seasonality	Exon	g10475	AT4G14200	Pentatricopeptide repeat		0.74	synonymous change
Isothermality	Promoter	g22449	AT5G35220	ammonium overly sensitive 1		0.75	n/a
Isothermality	Exon	g19730	AT4G01985	Unknown function		0.76	G to A
Isothermality	Intron	g7337	AT3G16460	jacalin-related lectin 34		0.74	n/a
Annual mean temperature	Intron	g10072	AT4G01985	Unknown function		0.71	n/a
Altitude	Intron	g35766	AT4g00360	SAM methyltransferase		0.76	n/a

The *Arabidopsis* homologs were determined from the TAIR/NCBI gene functions. *RDA* L values for redundancy analysis (RDA) loadings.

## Discussion

Our population genomic study of rewarewa (*Knightia excelsa*) has identified clear genetic and geographic structuring across Aotearoa New Zealand. Four different genetic clusters were identified across the complete geographic range of rewarewa. These genetic clusters were located in northern North Island (NNI), eastern North Island (NIE), western and southern North Island (NIWS) and the South Island (SI). Reconstruction of the population history revealed bottleneck and contraction events within these genetic clusters since the mid to late Pleistocene. The patterns revealed no evidence for human-mediated dispersal of the species, and we were able to identify several SNPs that were under selection and associated with environmental parameters. Additionally, we have complemented the rewarewa reference genome^10^ with a geographically comprehensive, and genome-wide variant and transcriptomic database, providing a substantial increase in the genomic resources available for this species, but also for the family Proteaceae more generally. This resource can be used to better understand the chemotypic properties of rewarewa honey, inform best practice for eco-sourcing when including rewarewa in restoration plantings, confirm its taxonomic placement and identification of close relatives, and supplement our understanding of the evolutionary history of an important member of Aotearoa New Zealand flora. This is the first population genetic study of rewarewa, the first genome-wide variant study for Proteaceae, and only the second study to apply pooled whole genome re-sequencing to a native Aotearoa New Zealand tree species.

Of the four genetic clusters found in rewarewa, the NNI and NIE genetic clusters were identified as being most closely related, whilst NNI and SI were found to be the most distantly related. We identified particularly strong genetic differentiation between the North and South Island populations across all analyses, with the South Island populations consistently forming their own cluster, separated from the North Island populations. The greatest mean pairwise *Fst* genetic distance was between SI and NNI (mean pairwise *Fst* = 0.334). Although some migration was identified between the North and South Island populations, this appears to be unidirectional (South Island to North Island) and was with the NIE cluster not with the NIWS cluster, the latter which is geographically closer to the South Island cluster. Additionally, of the four models used to explore the demographic history of divergence and gene flow between North and South Island populations (using SI and NIWS), the divergence followed by isolation (D-I) model was found to best explain the evolutionary history of these populations—not the models that allow for migration. This suggests there has been, and continues to be, a barrier to gene flow between the SI and NIWS genetic clusters—despite their geographic proximity.

There are a number of known biogeographical boundaries in New Zealand, and interestingly, genetic clustering within species often aligns with these boundaries^15^. Cook Strait, between the North and South Islands, is recognized as a natural barrier contributing to patterns of genotypic variation in many New Zealand tree species^26^. Another biogeographic boundary separates Northland from the rest of the North Island, which we see in the NNI genetic cluster. Thus, the NNI and SI genetic clusters are separated according to recognized biogeographic boundaries, but the split between the NIWS and NIE clusters is not a commonly seen pattern, and does not match any known biogeographic boundaries^26^. A similar geographical pattern of genetic structure was identified in the Aotearoa New Zealand native shrub mānuka (*Leptospermum scoparium*)^27^. Here, the same pool-seq method was applied, leading to the identification of five genetic clusters across Aotearoa New Zealand. Similar, genetic clusters were identified in Northland and the top of the South Island; however, the central North Island was dominated by one large genetic cluster, with a smaller genetic cluster isolated in the East Cape of the North Island. The barriers to gene flow that divided the genetic clusters of central North Island in mānuka appear to be different to those that form the western and eastern divide we see in this study, despite sharing similar colonization characteristics (e.g., following fire/disturbance events) and similar seed dispersal mechanisms (wind dispersal^5^).

The divergence between North and South Island rewarewa clusters was estimated to have occurred ~230,000—115,000 years ago, i.e., during the Pleistocene, (assuming a generation time of 5–10 years, which is based on the time it takes for a young rewarewa tree to achieve maturity and flowering). Our analyses also revealed gene flow between the SI and NIE genetic clusters, and bottleneck and contraction events within the genetic clusters, all occurring since the mid-late Pleistocene. The bottleneck in the South Island genetic cluster is estimated to have occurred between ~1.4 mya and ~580,000 years ago. There are fossil records that show rewarewa was previously more widely distributed in the South Island during the mid-late Pliocene to early Pleistocene (e.g., macro fossils at Arapito Rd, Karamea 3.5–1.81 mya; Waitahu River, Reefton 3.5–2.5 mya^28^. The bottleneck and population size contractions we inferred, especially in the South Island, may, therefore, be associated with decreases in geographic range. During the Pleistocene (2.58 million to 11,700 years ago) Aotearoa New Zealand was undergoing a period of climate cycling with glacial advances and retreats, leading to sea level rise and fall altering the coastline multiple times in different ways, and mountain building due to tectonic forces and volcanic activity^29^. The effect of this disturbance on the geographic distributions and patterns of genetic diversity in Aotearoa New Zealand species has been profound^27,30,31^.

Looking further back in time, a dated molecular phylogeny has shown that *Knightia* diverged from its sister taxon *Helicia* (Australia/SE Asia) and *Hollandaea* (Australia) 45.4 million years ago (±9.1 million years)^32^. However, Barker et al. cautioned that the inferred phylogenetic position of *Knightia* was dependent on only one gene. Nevertheless this date is much later than the rifting of Aotearoa New Zealand from Gondwana^33^ and therefore, the ancestor of *Knightia excelsa* likely dispersed to Aotearoa New Zealand. There is also evidence that around this time Proteaceae were more abundant and widespread throughout Aotearoa New Zealand, particularly during the Paleogene and early to mid Eocene (65–41.3 mya)^34,35^. However, without any extant close relatives in Aotearoa New Zealand, it is difficult to reconstruct the process of adaptation to the Aotearoa New Zealand environment.

Genetic distances between all clusters, but particularly North Island and South Island clusters, were relatively high for populations of the same species. Mean pairwise *Fst* estimates between NNI, NIE and SI were greater than 0.3, and between NIWS and SI it was >0.27. There are few pool sequencing analyses to utilize pairwise *Fst* for comparative purposes; however, if we assume that pairwise *Fst* values calculated in microsatellite studies will give similar values to those of pool sequencing studies, we can see that even considering its life history, rewarewa has relatively high pairwise *Fst* values^36^. For example, the pairwise *Fst* value for long-lived perennials is 0.19; for plants with mixed breeding systems (both outcrossing and selfing) it is 0.26; and for plants with wind-dispersed seeds, it is 0.13^36^. However, when successional status is considered, rewarewa appears to be more typical, as early successional plant species have pairwise *Fst* values of 0.37. This could be a result of early successional plants, usually being annual or short-lived perennials with the ability to self-fertilize, leading to a higher pairwise *Fst*. However, it is important to consider that there may be other factors that contribute to early successional species having higher pairwise *Fst*, even if they do not share those traits.

One pool-seq analysis we can compare our results to is that of mānuka^27^. The level of genetic differentiation between the North and South Island populations is comparable to the differentiation between Aotearoa New Zealand and Tasmanian *Leptospermum scoparium* (mean pairwise *Fst* = 0.325)^27^, which are separated by 2300 km of Tasman Sea (cf. with 22 km of Cook Strait between the North and South Island) and are considered differentiated enough to be reclassified as separate species^37^.

However, at this stage it appears unlikely that the North and South Island populations of rewarewa are sufficiently differentiated to merit species or even subspecies rank. Species delimitation requires a species concept, and evidence to assess species boundaries. Using the general lineage concept of de Queiroz et al., which considers species as separately evolving metapopulation lineages^38^, all available lines of evidence should be considered and synthesized when making taxonomic decisions. Currently, there is no data to suggest that genetic differentiation has led to reproductive isolation or morphological differentiation, but it may be worth collecting morphological data to explore this further. Due to the long narrow shape of Aotearoa New Zealand, there is a high likelihood of ecological differentiation between the North and South Island populations given climatic variables (e.g., annual precipitation and mean average temperature) vary significantly between the top of the North Island and the top of the South Island. In order to use ecological data as a justification for delimiting species, the range of the two putative species must be overlapping^39^. This is because if their ranges are not overlapping, as we have here with the NI vs SI populations of rewarewa, it is not possible to discount the null hypothesis of a single species with a broad ecological niche. Thus ecological data are unlikely to be useful for making taxonomic decisions in this case.

The GEA provides some novel knowledge on the genes linked to adaptation to environmental conditions in rewarewa. The RDA and LFMM analyses were used to establish the relationship between the identified SNPs (and nearby gene-coding sequences) and environmental variables. RDA is an unconstrained, multivariate ordination technique, and LFMM is a univariate GEA method. There are pros and cons to both methods, for example, LFMM can correct for confounding effects of population structure, however, LFMM can only process one environmental variable at a time^25^. RDA can analyze many loci and environmental variables simultaneously but is limited by its assumption of linear dependence between response and explanatory variables^40^; however, both methods have been found to be more powerful at detecting SNPs under selection, when compared to the likes of pcadapt (i.e., a method based on PCA)^41^. Both methods are subject to false discoveries, therefore, to reduce the False Discovery Rate (FDR), we used standard deviation of 2.5 and 3, and *Q* values of 0.1 and 0.05 as thresholds of statistical significance for the RDA and LFMM outputs, respectively. To further ensure the rigor of the significant SNPs identified, we then chose to only investigate SNPs that were identified as significant by both analyses. To increase the likelihood of identifying SNPs that were located within predicted gene models, we opted to investigate SNPs that met the less stringent thresholds of *q* value < 0.05 for the LFMM and 2.5 SD for the RDA (389 SNPs). Interestingly, over 42,000 SNPs were identified as significant by the RDA (c.f. 8116 SNPs for the LFMM), with >25% of these SNPs being associated with mean annual temperature. This may be due to most populations clustering at lower values for this variable, with the full range of values not being well represented across populations.

The detection of 386 significant SNPs across both analyses allowed us to identify patterns of genetic change linked to particular environmental conditions and formulate further hypotheses about the biological mechanisms involved in the adaptation to these variables. For example, *g5962*, a homolog of a gene belonging to the *O-fucosyltransferase* family of proteins, was linked to mean diurnal range. An *O-fucosyltransferase* called *SPINDLY* (*SPY*) negatively regulates the gibberellin signalling pathway in *Arabidopsis*^42^. Gibberellins are a type of plant hormone known to have a role in controlling plant growth, such as germination, flower development, and flowering time. Furthermore, *g35014*, which is also linked to the mean diurnal range, is a homolog of a gene belonging to the E3 ubiquitin ligases protein family that governs seed dormancy, germination, flowering and abiotic stress responses^43^. We also identified candidate genes that could be involved in rewarewa’s response to seasonal precipitation changes. For example, one of the precipitation/seasonality-associated genes is a homolog of *PENTATRICOPEPTIDE REPEAT* (*PPR*) superfamily gene. This gene family in *Arabidopsis* regulates drought, salt and cold stress responses^44^. The two isothermality-related SNPs were in those genes that shared sequence similarity with *ammonium overly sensitive 1* (*AMOS1*) and *jacalin-related lectin activity* (*JAL34*), which regulate environmental responses^45,46^. For altitude, an association was found for *g35766*, a gene homologous to *S-adenosylmethionine-dependent methyltransferase*. *S-adenosylmethionine dependent methyltransferases* regulate various stress responses in plants, such as oxidative stress^47,48^. Overall, these results offer valuable insight in understanding the genetic basis of various traits in rewarewa and may serve as a basis for functional characterization and potential applications in breeding programs.

We have developed a new understanding of the population structure of rewarewa in Aotearoa New Zealand: demographic history and insights into the genetic control of adaptive traits will have significant impact on predicting the species’ potential adaptation to climate change and will be a valuable resource for informing restoration plantings. The set of DNA variants identified and the genes predicted are useful resources to mine loci linked not only to adaptive traits, but also to traits that may be beneficial to the honey industry, for example, the antioxidant content in rewarewa honey, derived from its nectar chemistry. Furthermore, the knowledge of the genetic structure of rewarewa has enabled the delimitation of regional provenances, enabling rewarewa honey producers, including Māori agribusinesses to consider commercializing their honey based on its origin.

## Methods

### Sample collection

**Fig. 7:**
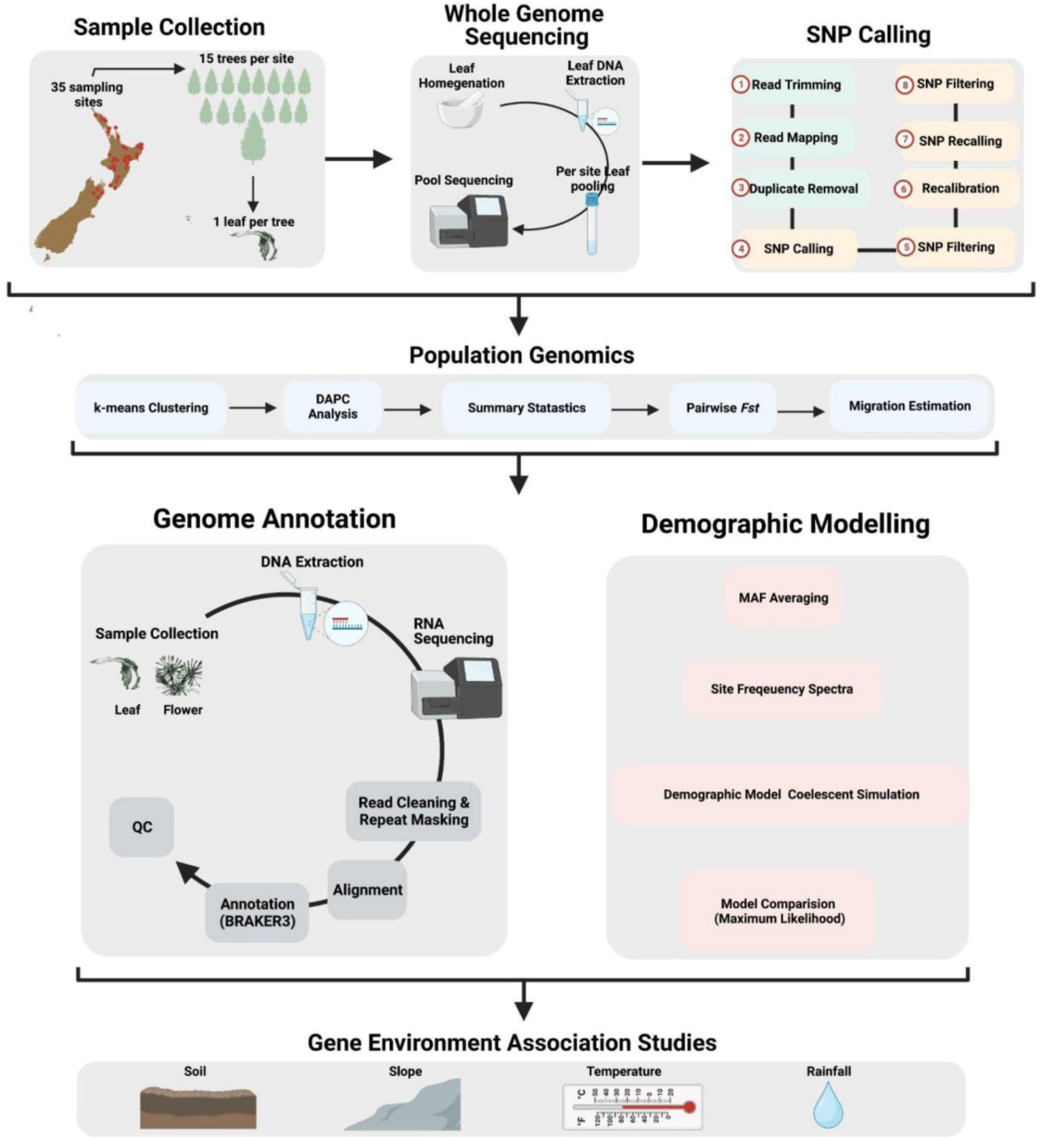
Overview of the rewarewa population genomics workflow. The workflows used for sampling, whole genome sequencing, single nucleotide polymorphism (SNP), population genomics, gene annotation, demogrphic modelling and gene environment association (GEA) analysis are described.

The experimental procedure is illustrated in Fig. 7. The geographic range of rewarewa was identified by mapping georeferenced samples from the herbaria CHR, AK, WELT (herbarium acronyms follow Index Herbariorum, see http://sweetgum.nybg.org/ih/, accessed 18 April 2023) and the National Vegetation Survey database (NVS). Between 2017–2019 samples (leaves) from 35 populations across the geographic range were collected using pole pruners (Supplementary Table 8). At each sampling location, leaves from 15 mature trees were collected into silica, and a voucher was collected and accessioned at CHR. In a few cases the mature trees were too tall for the long-handled pole pruners to reach, and so instead, leaves were obtained from a sapling that was growing underneath a mature tree. Collections on land managed by the New Zealand Department of Conservation (15 sites) were collected under permit number CA-31615-OTH, and for 12 of these sites, consultation was undertaken with relevant Māori iwi (tribes) or hapū (sub-tribes). Four sampling sites on council-managed land were collected with permission from the Auckland Council in consultation with relevant iwi, the Greater Wellington Regional Council and Horizons (Manawatū Regional Council). The remainder of the populations (16 sites) were collected from private land, including 10 sites consented by Māori landowners.

### DNA extraction

Genomic DNA was extracted from the nucleus using Macherey-Nagel NucleoSpin(R) Plant II Kit. Dried leaves of rewarewa (1 cm^2^) were homogenized in liquid nitrogen using a ceramic mortar and pestle. From here, leaves were further processed as per the kit protocol with the following modifications: the volume of PL2 buffer was increased to 400 μL, and 13.3 μL of Proteinase K and 13.3 μL of RNaseA was added at the start, after 1 h and 1.30 min of 2 h incubation at 57° C. The quality of the DNA was assessed using a standard 1% agarose gel electrophoresis separation, and samples that showed degraded DNA below 10,000 kb were excluded. A fluorometer was used to quantify the volume of DNA (Qubit high sensitivity dsDNA kit). A minimum of 20 ng/μl was obtained for each sample, with the average yield of nuclear genomic DNA per gram of leaf sample being 1 µg. Across each of the 35 sample sites, 15 samples per site were pooled, and each pool had a concentration of 20 ng/μl. The pooled DNA was sent to the Australian Genome Research Facility Limited (AGRF) for whole genome sequencing.

### Variant detection analysis

Reads were downloaded and quality assessed using FastQC (Version 0.11.7)^49^. After inspection, the reads were filtered using TrimGalore (Version 0.6.4) (https://github.com/FelixKrueger/TrimGalore) utilizing the –nextseq function (./trim_galore –nextseq 16 –paired $input.fastq.gz -o $input_trimmed/). After trimming, each population read set was mapped using BWA (Version 0.7.17)^50^ to the pseudo-chromosomal rewarewa assembly (./bwa mem -P -M reference.fasta <Forward_readset1.fq > <Reverse_readset1.fq > > population_Flye.sam). Files were compressed using the SAMtools (Version 1.10)^51^ view function, sorted by position and read groups added using the picard (Version 2.21.8-Java-11.0.4) (http://broadinstitute.github.io/picard/) AddOrReplaceReadGroups functionality (samtools view -b population_Flye.sam > population_Flye.bam | picard AddOrReplaceReadGroups I=population_Flye.bam O=population_Flye_sort.bam SORT_ORDER=coordinate RGID = <populationID> RGLB=Rewa_PoolSeq RGPL=illumina RGPU = <populationIDinfo> RGSM=Rewa_Pool). After this, duplicated reads were flagged for subsequent identificationusing picard MarkDuplicates function and indexed using SAMtools faidx (picard MarkDuplicates I=population_Flye_sort.bam O=population_Flye_sort.bam M=population_Flye_sort_metrics.txt OPTICAL_DUPLICATE_PIXEL_DISTANCE = 2500 CREATE_INDEX=true).

A sequence dictionary was created for the rewarewa reference genome utilizing picard’s CreateSequenceDictionary function, and was subsequently indexed using SAMtools faidx command (./picard CreateSequenceDictionary R= reference.fasta O= reference.dict | ./samtools faidx reference.fasta). Variant detection was carried out on all indexed bam files using GATK (Version 4.1.8.1)^12^ HaplotypeCaller function (gatk HaplotypeCaller -R reference.fasta -I population_Flye_sort.bam –sample-ploidy 60 –max-genotype-count 61 -O population_Flye_sort.vcf). Sample-ploidy was then calculated by using 2 x pool size and the max-genotype parameter was calculated using the (F[P,A]) formula, where P= ploidy and A=Allele count. All other variant classes were excluded through GATK SelectVariants by implementing the –select-type-to-include parameter (gatk SelectVariants -R reference.fasta -V population_Flye_sort.vcf -O population_Vars.vcf –select-type-to-include SNP).

Identified low-quality SNPs were removed. We first generated a table of variant quality using GATK VariantsToTable function in order to determine the hard-filtering parameters required. In-house R scripts were written (Supplementary Information Text) to generate summary statistics from each population’s variant table: density plots of each summary statistic were then generated in order to visually inspect cut-off points for each parameter. Once filtering parameters had been determined, filtering was carried out using GATKs VariantFiltration function (gatk VariantFiltration -R reference.fasta -V population_Vars.vcf -O population_filtration.vcf -filter “QUAL < X” –filter-name Low_Qual -filter “DP < X” –filter-name Low_Cov -filter “QD < X || MQ < X || FS > X || SOR > X || MQRendSum < -X” –filter-name Secondary_filter). In order to remove the reads that failed hard-filtering parameters, the GATK SelectVariant function was also run (gatk SelectVariants -R reference.fasta -V population_filtration.vcf -O population_postfiltration.vcf –exclude-filtered).

In accordance with GATK guidelines, all base quality scores were recalibrated after duplicate removal, as a ‘true SNP’ set was required. For this, each population’s filtered SNP calls (population_postfiltration.vcf) were utilized as bootstrapping the recalibration of the original bam file with duplicates removed (population_Flye_sort.bam). This recalibration was done in two steps by firstly generating a recalibration table through GATKs baseRecalibration tool (gatk BaseRecalibrator -I population_Flye_sort.bam -R reference.fasta -O population_recalibrated_table –known-sites population_postfiltration.vcf) which uses the GATKs ApplyBQSR tool for the actual recalibration (gatk ApplyBQSR -R reference.fasta -I population_Flye_sort.bam –bqsr-recal-file population_recalibrated_table -O population_recalibrated.bam).

After recalibration, SNP calling was re-run utilizing GATKs HaplotypeCaller to produce a set of recalibrated filtered SNP calls in both bam and vcf formats (gatk HaplotypeCaller -R reference.fasta -I population_recalibrated.bam –sample-ploidy 60 –max-genotype-count 61 -O population_recalibrated_SNPcalls.vcf –bam-output population_recalibrated_SNPcalls.bam).

Stringent filtering was applied to the raw vcf file in vcftools v0.1.14, filtering for biallelic SNPs only (--min-alleles 2 –max-alleles 2); a mean depth of 100 (--min-meanDP 100); no missing data (--max-missing 1) and a minor allele frequency (MAF) of 0.05.

### Population genomics

Minor allele frequencies were calculated using the R^52^ package vcfR v1.8.0^53^. Following this, the package Adegenet v2.1.1^13,14^ was used to explore the population structure of the rewarewa samples. First, a k-means clustering analysis (find.clusters function) was carried out to determine the optimal K value (optimal number of ancestral populations) (*n* = 30, n.pca = 35, scale = FALSE, choose.n.clust = FALSE, criterion = “min”). Subsequently a DAPC analysis was run (n.da=100, n.pca=optim.a.score) using this optimized K value, in addition to an optimized number of principal components (PCs) (optim.a.score function). Summary statistics and neutrality tests including number of segregating sites (*S*), nucleotide diversity (*π*), Watterson’s θ and Tajima’s D were estimated using NPStats v1^18^ (-n 30 -l 10,000 -mincov 25 -maxcov 500). Pairwise Fixation Indices (*F*st) were estimated using PoolFstat v1.0.0^54^ (method = “Anova”). SplitsTree4 v4.14.8^16^ and ade4 v1.7-15^17^ were used to explore the pairwise *F*st output. Migration between populations was investigated using TreeMix v1.13^19^ (-k 1000) where 31 migration events (1–30) were explored for five iterations. As the South Island populations were determined to be genetically distinct from the North Island populations in our prior analyses, they were used to root the TreeMix phylogenies. OptM v0.1.6^20^ was used to determine the number of TreeMix migration events that best explained the variance within the dataset (method = “Evanno”, thresh = 0.05).

**Fig. 8:**
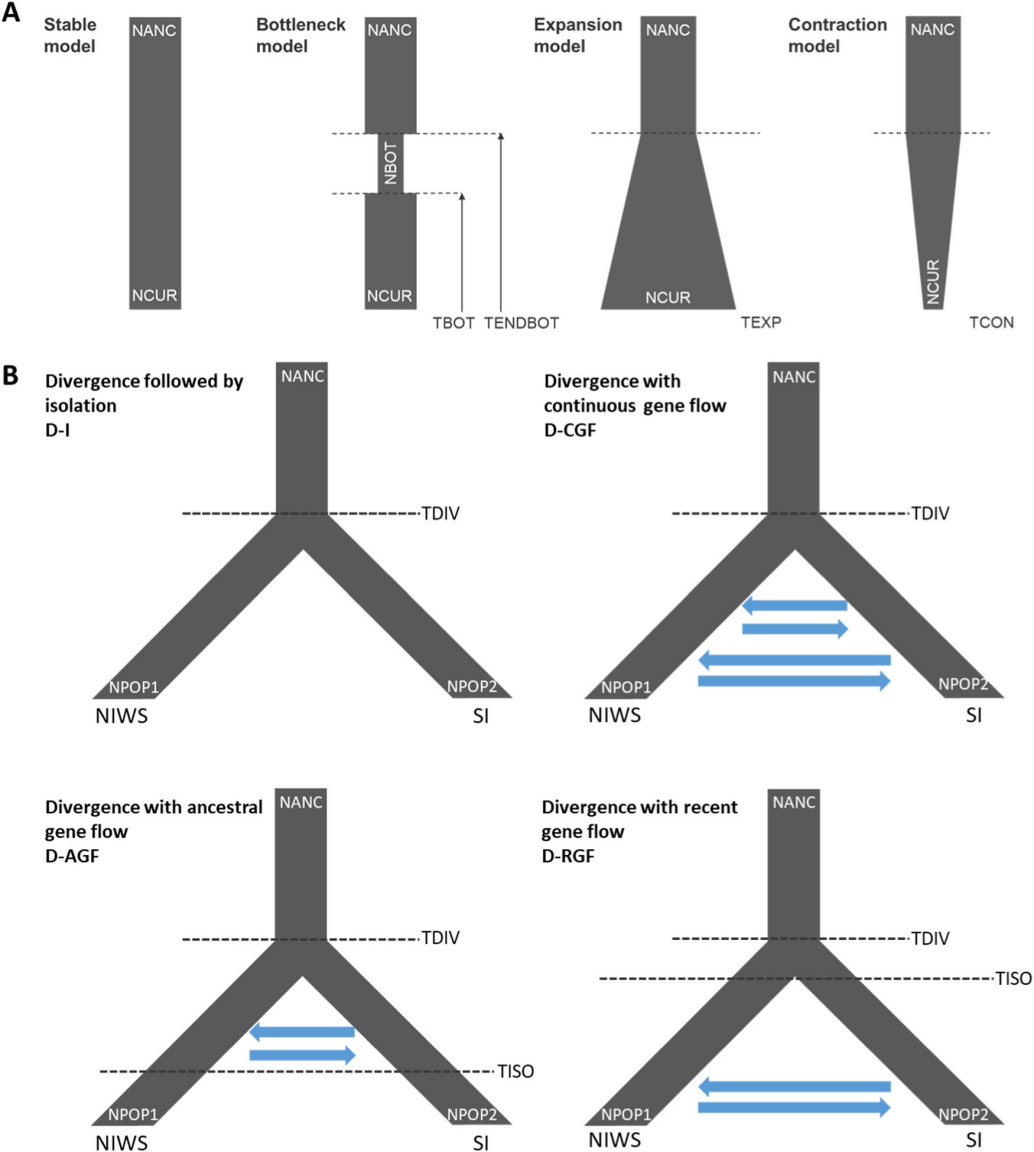
Diagram of demographic models, modified from Koot et al.^27^. NANC effective population size of ancestral population. NCUR effective population size of current population, NBOT effective population size at time of bottleneck, NPOP1 current effective population size population one, NPOP2 current effective population size population two, TBOT time of bottleneck, TENDBOT time of end of bottleneck, TEXP time of expansion, TCON time of contraction, TDIV time of division, TISO time of isolation, SI South Island, NIWS western and southern North Island. Arrows indicate the direction of migration. **A** Diagram of demographic models tested on individual genetic clusters. **B** demographic models were used to estimate the split of North and South Island genetic clusters.

The demographic history of the genetic clusters was explored using the coalescent simulation-based method fastsimcoal v2.6.0.3^21^. Minor allele frequencies were averaged across genetic clusters, from which folded site frequency spectra (SFS) were calculated using SweepFinder2^55^ and a custom R script^27^. The SFS scores were then used to explore the demographic histories of individual genetic clusters, and a 2D SFS matrix between South Island and Western North Island genetic clusters estimation conducted to explore the demographic history of divergence and gene flow between North and South Island populations. Four hypotheses were tested in investigating the demographic history of each genetic cluster: stable, expansion, contraction and bottleneck (Fig. 8)^27^. Additionally, four hypotheses were tested for the history between the North and South Island: divergence followed by isolation (D-I); divergence followed by continuous gene flow (D-CGF); divergence with ancestral gene flow followed by isolation (D-AGF-I); divergence with only recent gene flow (D-RGF) (Fig. 8)^27^. For each model, 100,000 coalescent simulations were applied, with maximum likelihood estimates calculated based on differences between the input observed SFS and the output expected SFS. Models were repeated 100 times, a global maximum likelihood estimate was obtained from these independent runs, and Akaike’s Information Criterion (AIC) calculated for model comparison and selection.

### Genotype environment association

Environmental layers covering various characteristics of each population, including soil, slope, temperature and rainfall, were obtained from the Worldclim website (https://worldclim.org/data/ worldclim21.html, accessed 19 August 2021^56^) and Land Environments New Zealand (LENZ; https://lris.scinfo.org.nz/, accessed 19 August 2021^57^), for details see Supplementary Table 9. To co-analyze the LENZ and WorldClim layers, the resolution and projection of the LENZ layers were transformed to match those of the WorldClim data. This was undertaken in R (ver. 3.1.3, R Foundation for Statistical Computing, Vienna, Austria) and RStudio (ver. 0.97, RStudio, Inc., Boston, MA, USA, see http://www.rstudio.com, accessed 15 April 2021), using the function spatial_sync_raster from the package spatial.tools (ver. 1.4.8, J. A. Greenberg, see http://CRAN.R-project.org/ package=spatial.tools). Principal Component Analysis (PCA) plots and Pairwise Pearson’s ‘r’ correlation coefficients were used to check for collinearity between variables using the packages factoextra v1.0.7^58^ and Hmisc v4.5-0^59^, respectively. Genotype Environment Associations (GEAs) were estimated using Redundancy Analysis (RDA) in Vegan^40^ and LFMM in lfmm2^25^. For the RDA, standard deviations of 2.5 and 3, and for the lfmm2, Q-values of 0.1 and 0.05 were explored as thresholds of statistical significance. Q-values were calculated using the *q* value package^60^. Variants that met this threshold for both RDA and LFMM analyses were considered to putatively demonstrate a significant GEA.

### Annotation

RNA was obtained from one to two flowers and leaves of rewarewa plants sourced from Riverland Nurseries Limited, Auckland, New Zealand. The fresh tissues were snap-frozen in liquid nitrogen, and 2–3 µg RNA with a RIN value > 8. 0 was extracted and purified utilizing Spectrum™ Plant Total RNA Kit (STRN50) according to manufacturer’s instructions. The RNA samples were subjected to DNase digestion using an on-column DNase I digest set, catalog no. DNASE70. The total RNA library was prepared and sequenced on NovaSeq SP by AGRF.

From the flower and leaf samples collected, four flow cells, totaling 15.46 Gb of 150 bp paired end RNASeq reads were generated. The reads were quality checked using the fastqc software package v0.11.9^49^. Results indicated the presence of an Illumina Universal Adaptor sequence; these were subsequently removed using trimgalore v0.6.7^61^ (trim_galore –paired –illumina -j 2 flowers_HJWC5DSX3_TGGCCGGTAT-GCGCTCTAAT_L003_R1.fastq.gz flowers_HJWC5DSX3_TGGCCGGTAT-GCGCTCTAAT_L003_R2.fastq.gz). Although the first 50 bp appeared high in duplicated sequence, this is a common feature of RNAseq data as some transcripts are more abundant than others. Thus, some transcripts may appear so abundant that they register as an overrepresented sequence. In this case, the first 50 bp had adequate Phred scores, and a normal GC content, so to maximize read length, this sequence was not removed.

To prepare the reference genome for annotation, repeats first needed to be identified and masked where possible. To do this, Repeatmodeller version open-1.0.8^62^ and Repeatmasker v4.1.2-p1 (http://www.repeatmasker.org) were employed, respectively (BuildDatabase -name rewarewa_repeatmodelerDB <reference_genome_file> | RepeatModeler -database Rewa -pa 8 -LTRStruct >& run.out & | RepeatMasker -pa 8 -gff -nolow -lib consensi.fa.classified <fasta_file>).

The reads were aligned to the reference genome using the most recent STAR aligner version 2.7.10b^22^. To prepare the reference genome, it first needed to be indexed (STAR –runThreadN 6 –runMode genomeGenerate –genomeDir <index_file > –genomeFastaFiles <reference_genome_file > –genomeSAindexNbases 13). Mapping was conducted (/public/groups/cgl/local/bin/STAR –runThreadN 8 –genomeDir <directory_name > –outFileNamePrefix <prefix_preference > –readFilesIn <reads_file > ) and the alignment converted to BAM format and sorted using samtools version 1.9^51^ (samtools view –threads 8 -b -o <bam_file > <sam_file > >& <output_file> & |samtools sort -m 7 G -o <sorted_bam > -T <temp_file > –threads 8 <bam_file > >& r<output_file> &). Alignment quality metrics were generated using Picardtools (picard CollectAlignmentSummaryMetrics REFERENCE_SEQUENCE = <reference_genome_file> INPUT = <sorted_bam> OUTPUT = <output_file > ).

After inspection, GeneMark-ES/ET/EP and its license key were downloaded and installed according to GeneMark’s author instructions, and a BRAKER3 version 3^23^ singularity container was built and executed using the “singularity exec braker.sif braker.pl” command. Annotation was conducted using BRAKER3 (singularity shell -B /BRAKER/ braker.sif braker.pl –species=Rewarewa –genome = <masked_reference_genome > –bam = <sorted_bam > >& <log_file> &), the resulting .gtf file was run through BUSCO version 5.4.4^63^, using both the eukaryotic and eudicot database (./busco –in euk_coding.fa –out euk_busco -m tran). The annotation pipeline and quality control analysis were implemented in nextflow and made publicly available (https://github.com/kherronism/rewarewaannotation).

A comprehensive examination of the identified SNPs in the context of promoters, introns, and exons was performed to identify genes linked to the traits of interest. To confirm the accuracy of results, we cross-checked the SNPs with the annotated rewarewa genome. Using Geneious Prime (https://www.geneious.com/), we searched the positions of the SNPs in the genome and visualized their genomic context. For each SNP, its location within the gene structure, such as promoter regions, introns, or exons, was determined. The potential functional impact of the SNPs located within coding regions was examined to investigate whether they caused amino acid changes or not. This was achieved by comparing the reference and alternative alleles at each SNP position and translating the corresponding coding sequence into amino acids. The comparison of amino acid sequences before and after the SNP allowed the identification of non-synonymous SNPs (causing amino acid changes) and synonymous SNPs (producing no change to amino acid sequence). The potential candidate genes associated with the traits of interest were identified by combining information on the genomic context and the potential functional impact of the SNPs.

### Indigenous data sovereignty

The research was co-developed alongside Māori agribusiness partners in the Aotearoa New Zealand honey sector, who have an interest in developing regional branding associated with their traditional connections to the land. A Māori governance rōpū (group) was established to provide research project oversight as well as to coordinate access to plant material and engagement with Māori land owners. Samples for sequencing and downstream analysis were collected from natural stands of rewarewa, some grown on Māori freehold land (24 out of 35 populations—see Sample Collection section above). Prior to commencing the research, Māori with historical and territorial rights over this land were individually approached and informed about the proposed research objectives. Consent to access the plant material was granted for the purposes of the research study. Reconsenting was also explicitly requested if (1) access to more samples was required; (2) any further analysis was to be conducted on the previously collected samples; and (3) any subsequent research would potentially disclose the exact identity of the trees.

To respect the terms and conditions of this consent and recognize the rights of Indigenous Peoples and their sovereignty^64,65^ that have been codified in the United Nations Declaration on the Rights of Indigenous Peoples (UNDRIP), all sampling sites have been generalized to only reflect the region with both the exact location of the samples collected, and identity of the Indigenous Peoples who contributed, being purposefully redacted.

### Supplementary information

Supplementary Table 2

Supplementary information

## Data Availability

All raw and assembled transcriptomic and pooled sequencing data are stored in the Aotearoa Genomics Data Repository (AGRD; https://www.genomics-aotearoa.org.nz/data).
