# Supplementary material for: A population genomics analysis of the Aotearoa New Zealand endemic rewarewa tree (Knightia excelsa)
Source: NPJ Biodivers. 2024 Mar 20;3:7. doi: 10.1038/s44185-024-00038-6 (PMC11332057; doi:10.1038/s44185-024-00038-6)
Supplement: Supplementary file 2 — Supplementary information [file 44185_2024_38_MOESM2_ESM.docx]

**A population genomics analysis of the Aotearoa New Zealand endemic rewarewa tree (*Knightia excelsa*)**

Ann M. McCartney^1,2,6†^, Emily Koot^2,5†^, Jessica M. Prebble^4^, Rubina Jibran^7^, Caroline Mitchell^4^, Ana Podolyan^4^, Alex Fergus^4^, Elise Arnst^4^, Taina Goldsmith^??^, Gary Houliston^4^, Thomas R. Buckley^1,2,3^, David Chagné^2,5^

**Supplementary Figure 1: Sampling locations of each population used in the population genomic study.** Points are coloured by gene pool. Points are purposefully enlarged to address the sensitivity of this indigenous intellectual property.

**
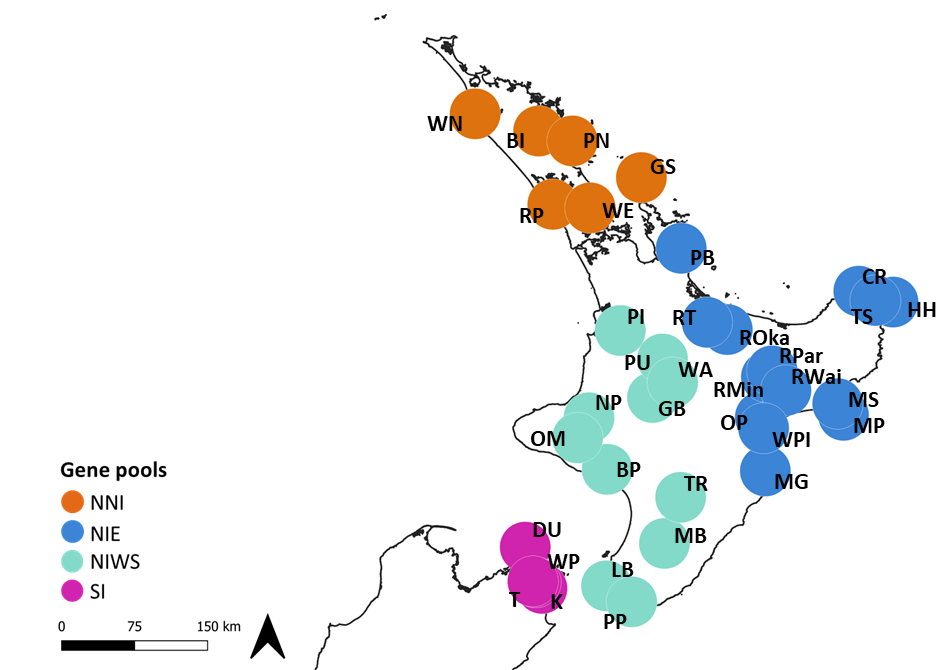
**

**Supplementary Scripts**

**Supplementary Scripts 1: Variant Calling**

**# Initial Mapping of Pool-Sequencing populations to Reference Genome:**

ml BWA

bwa mem -P -M FLYE_genome.fasta X.fq.gz X.fq.gz > BI_Flye.sam

**# Bam sorting**

ml SAMtools

ml picard/2.21.8-Java-11.0.4

export _JAVA_OPTIONS=-Djava.io.tmpdir=X

samtools view -b BI_Flye.sam > BI_Flye.bam

picard AddOrReplaceReadGroups I=Flye.bam O=Flye_sort.bam SORT_ORDER=coordinate RGID=HW33MDSXX RGLB=Rewa_PoolSeq RGPL=illumina RGPU=HW33MDSXXGTTCCAATAATTCTGC RGSM=Rewa_Pool

**# Duplication Removal**

ml SAMtools

ml picard/2.21.8-Java-11.0.4

export _JAVA_OPTIONS=-Xmx64G

export _JAVA_OPTIONS=-Djava.io.tmpdir=X

picard MarkDuplicates I=Flye_sort.bam O=Flye_sort_duprem M=duprem_metrics.txt OPTICAL_DUPLICATE_PIXEL_DISTANCE=2500 CREATE_INDEX=true

**# Variant Detection**

ml SAMtools

ml picard/2.21.8-Java-11.0.4

ml GATK/4.1.4.1-gimkl-2018b

export _JAVA_OPTIONS=-Xmx64G

export _JAVA_OPTIONS=-Djava.io.tmpdir=X

gatk HaplotypeCaller -R FLYE_genome.fasta -I Flye_sort_duprem --sample-ploidy 60 --max-genotype-count 61 -O population1.vcf

**# SNP_Subsetting**

ml SAMtools

ml picard/2.21.8-Java-11.0.4

ml GATK/4.1.4.1-gimkl-2018b

export _JAVA_OPTIONS=-Xmx31G

export _JAVA_OPTIONS=-Djava.io.tmpdir=X

gatk SelectVariants -R FLYE_genome.fasta -V population1.vcf -O population1_Vars.vcf --select-type-to-include SNP

**# Make Variants Table prior to Hard Filtration**

ml SAMtools

ml picard/2.21.8-Java-11.0.4

ml GATK

export _JAVA_OPTIONS=-Xmx32G

export _JAVA_OPTIONS=-Djava.io.tmpdir=X

gatk VariantsToTable -R FLYE_genome.fasta -V population1_Vars.vcf -O NP_VarsToTable -F CHROM -F POS -F QUAL -F DP -F QD -F MQ -F FS -F SOR -F MQRankSum

*#Convert files to .csv format using simple unix 'tr' command*

*#Use attached R scripts written by me to generate summary stats and density plots. Assess stats and plots to inform hard filters.*

**#Apply Filters Variants based on stats and plots**

ml SAMtools

ml picard/2.21.8-Java-11.0.4

ml GATK/4.1.4.1-gimkl-2018b

export _JAVA_OPTIONS=-Xmx31G

export _JAVA_OPTIONS=-Djava.io.tmpdir=X

gatk VariantFiltration \

-R FLYE_genome.fasta \

-V population1_Vars.vcf \

-O population1_Vars_filtration.vcf \

-filter "QUAL < 20" --filter-name Low_Qual \

-filter "DP < 100" --filter-name Low_Cov \

-filter "QD < 1.0 || MQ < 50.0 || FS > 1.0 || \

SOR > 1.0 || MQRendSum < -20.0" \

--filter-name Secondary_filter

**# Generate file containing only SNPs passing hard filters**

ml SAMtools

ml picard/2.21.8-Java-11.0.4

ml GATK/4.1.4.1-gimkl-2018b

export _JAVA_OPTIONS=-Xmx31G

export _JAVA_OPTIONS=-Djava.io.tmpdir=X

gatk SelectVariants -R FLYE_genome.fasta -V population1_Vars_filtration.vcf -O population1_Vars_postfiltration.vcf --exclude-filtered

**# Generate recalibration table based on filtered variants**

ml SAMtools

ml picard/2.21.8-Java-11.0.4

ml GATK/4.1.4.1-gimkl-2018b

export _JAVA_OPTIONS=-Xmx31G

export _JAVA_OPTIONS=-Djava.io.tmpdir=X

gatk BaseRecalibrator -I Flye_sort_duprem.bam -R FLYE_genome.fasta -O population1_recalibrated_table --known-sites population1_Vars_postfiltration.vcf

**# Base Quality Score recalibration**

ml SAMtools

ml picard/2.21.8-Java-11.0.4

ml GATK/4.1.4.1-gimkl-2018b

export _JAVA_OPTIONS=-Xmx31G

export _JAVA_OPTIONS=-Djava.io.tmpdir=X

gatk ApplyBQSR -R FLYE_genome.fasta -I population1_Flye_sort_duprem.bam --bqsr-recal-file population1_recalibrated_table -O population1_recalibrated.bam

**# Recall your SNPs**

ml SAMtools

ml picard/2.21.8-Java-11.0.4

ml GATK/4.1.4.1-gimkl-2018b

export _JAVA_OPTIONS=-Xmx64G

export _JAVA_OPTIONS=-Djava.io.tmpdir=X

gatk HaplotypeCaller -R FLYE_genome.fasta -I population1_recalibrated.bam --sample-ploidy 60 --max-genotype-count 61 -O population1_recalibrated_SNPcalls.vcf --bam-output population1_recalibrated_SNPcalls.bam

**# Scripts for Statistics and Density Plot Generation to set Hard Filters**

*#Statistics Generator for Hard Filtering: Code written in R*

*#Written by Ann Mc Cartney*

*#Input: VarsToTable output from GATK workflow that has been converted to .csv format.*

*#Output: .txt file with Statistical analysis of Variants in Table.*

*#Used to guide hard filters to implement.*

library("ggplot2")

data <- read.csv("$i.csv", header=TRUE)

sink('Stats_$i_output.txt')

*("===================SUMMARY_STATS========================")*

*("============written by: ANN_MC_CARTNEY==================")*

*("========================================================")*

cat("MQ_STATS\n")

cat("Max:")

max(data$MQ,na.rm=TRUE)

cat("Mean:")

mean(data$MQ,na.rm=TRUE)

cat("=============================\n")

sink('Stats_$i_output.txt', append=TRUE)

cat("SOR_STATS\n")

cat("Max:")

max(data$SOR,na.rm=TRUE)

cat("Mean:")

mean(data$SOR,na.rm=TRUE)

cat("=============================\n")

sink('Stats_$i_output.txt', append=TRUE)

cat("MQRankSum_STATS\n")

cat("Max:")

max(data$MQRankSum,na.rm=TRUE)

cat("Min:")

min(data$MQRankSum,na.rm=TRUE)

cat("Mean:")

mean(data$MQRankSum,na.rm=TRUE)

cat("=============================\n")

sink('Stats_$i_output.txt', append=TRUE)

cat("FS_STATS\n")

cat("Max:")

max(data$FS,na.rm=TRUE)

cat("Mean:")

mean(data$FS,na.rm=TRUE)

cat("=============================\n")

sink('Stats_$i_output.txt', append=TRUE)

cat("QD_STATS\n")

cat("Max:")

max(data$QD,na.rm=TRUE)

cat("Mean:")

mean(data$QD,na.rm=TRUE)

cat("=============================\n")

sink('Stats_$i_output.txt', append=TRUE)

cat("QUAL_STATS\n")

cat("Max:")

max(data$QUAL,na.rm=TRUE)

cat("Mean:")

mean(data$QUAL,na.rm=TRUE)

cat("=============================\n")

sink('Stats_$i_output.txt', append=TRUE)

cat("DP_STATS\n")

cat("Max:")

max(data$DP,na.rm=TRUE)

cat("Mean:")

mean(data$DP,na.rm=TRUE)

cat("=============================\n")

sink('Stats_$i_output.txt', append=TRUE)

sink()

quit()

#Generate density plots from to guide Hard Filtration specification

#Written by Ann Mc Cartney

#Input: VarsToTable output from GATK workflow that has been converted to .csv format

#Output: .jpg files containing density plots of summary statistics.

#Required: ggplot2

#Used to guide hard filters to implement.

library("ggplot2")

data <- read.csv("$i.csv", header=TRUE)

# Basic MP

p <- ggplot(data, aes(x=DP)) +

geom_density()

p

# Add mean line

jpeg('DP.jpg')

p+ geom_vline(aes(xintercept=mean(DP)),

color="blue", linetype="dashed", size=1)

dev.off()

# Basic QUAL

p <- ggplot(data, aes(x=QUAL)) +

geom_density()

p

# Add mean line

jpeg('QUAL.jpg')

p+ geom_vline(aes(xintercept=mean(QUAL)),

color="blue", linetype="dashed", size=1)

# Basic QD

p <- ggplot(data, aes(x=QD)) +

geom_density()

p

# Add mean line

jpeg('QD.jpg')

p+ geom_vline(aes(xintercept=mean(QD)),

color="blue", linetype="dashed", size=1)

dev.off()

Column numbers

# Basic MQ

p <- ggplot(data, aes(x=MQ)) +

geom_density()

p

# Add mean line

jpeg('MQ.jpg')

p+ geom_vline(aes(xintercept=mean(MQ)),

color="blue", linetype="dashed", size=1)

dev.off()

Column numbers

# Basic FS

p <- ggplot(data, aes(x=FS)) +

geom_density()

p

# Add mean line

jpeg('FS.jpg')

p+ geom_vline(aes(xintercept=mean(FS)),

color="blue", linetype="dashed", size=1)

dev.off()

# Basic SOR

p <- ggplot(data, aes(x=SOR)) +

geom_density()

p

# Add mean line

jpeg('SOR.jpg')

p+ geom_vline(aes(xintercept=mean(SOR)),

color="blue", linetype="dashed", size=1)

dev.off()

# Basic MQRankSum

p <- ggplot(data, aes(x=MQRankSum)) +

geom_density()

p

# Add mean line

jpeg('MQRankSum.jpg')

p+ geom_vline(aes(xintercept=mean(MQRankSum)),

color="blue", linetype="dashed", size=1)

dev.off()

**Supplementary Scripts 2: Population genomics**

**# Merge VCF files**

module load VCFtools/0.1.14-gimkl-2018b-Perl-5.28.1

module load BCFtools/1.10.2-GCC-9.2.0

module load tabix

cd X

for VCF in *_SNPcalls.vcf;

do

bgzip $VCF

done

for VCF in *_SNPcalls.vcf.gz;

do

bcftools annotate -x INFO,FORMAT/GT,FORMAT/GQ,FORMAT/PL $VCF -Oz -o $VCF.filtered.vcf.gz

done

for VCF in *_SNPcalls.vcf.gz.filtered.vcf.gz;

do

tabix -f -p vcf $VCF

done

ls *_SNPcalls.vcf.gz.filtered.vcf.gz > vcfout.list

bcftools merge --force-samples --file-list vcfout.list -Oz -o Rewarewa_merged.vcf.gz

vcftools --gzvcf Rewarewa_merged.vcf.gz --min-alleles 2 --max-alleles 2 --min-meanDP 100 --remove-indels --recode --stdout | gzip -c > Rewarewa_merged_filtered2.vcf.gz

bcftools query -f '%CHROM %POS[\t%AD]\n' Rewarewa_merged_filtered2.vcf.gz > Rewarewa_AD_only.txt

sed 's/\,[0-9]*[^\t]//g' Rewarewa_AD_only.txt > Rewarewa_AD_Ref.txt

vcftools --gzvcf Rewarewa_merged_filtered2.vcf.gz --site-depth --out Rewarewa_site_depth

**# Calculate allele frequency for filtering**

library(data.table)

setwd("/Rewarewa/Analyses/")

vcf <- read.table("Rewarewa_AD_Ref.txt",header=FALSE, na.strings = ".")

vcf$V39<-rowSums(vcf[, c(3:38)])

depth <- read.table("Rewarewa_site_depth.ldepth",header=TRUE)

depth$REF_SUM <- vcf$V39

depth$ALT_SUM <- (depth$SUM_DEPTH - depth$REF_SUM)

depth$freq_ref<-(depth$REF_SUM/depth$SUM_DEPTH)

depth$freq_Alt<-(depth$ALT_SUM/depth$SUM_DEPTH)

depth$freq_maf<-ifelse(depth$freq_ref < 0.5,depth$freq_ref,depth$freq_Alt)

freq1<-depth %>% filter(depth$freq_maf > 0.05)

freq2<-depth %>% filter(depth$freq_maf < 0.05)

freq1$SUM_DEPTH <- NULL

freq1$SUMSQ_DEPTH <- NULL

freq1$REF_SUM <- NULL

freq1$ALT_SUM <- NULL

freq1$freq_ref <- NULL

freq1$freq_Alt <- NULL

freq1$freq_maf <- NULL

fwrite(freq1,"MAF_95_positions.txt", sep = "\t")

**# Filter SNPs based on MAF 0.05**

module load vcftools

vcftools --gzvcf Rewarewa_merged_filtered2.vcf.gz --positions MAF_95_positions.txt --recode --stdout | gzip -c > Rewarewa_merged_filtered_maf.vcf.gz

**# Create allele frequency file for analyses (transposed and without missing data)**

library(vcfR)

library(data.table)

setwd("/Rewarewa/Analyses/")

vcf <- read.vcfR( file = "/Rewarewa_merged_filtered_maf.vcf.gz", verbose = FALSE )

ad<-extract.gt(vcf, element = "AD")

freq1<-AD_frequency(ad, delim = ",", allele = 2L)

freq1<-as.data.frame(freq1, rownames=rownames)

miss<-freq1[!complete.cases(freq1),]

freq <- na.omit(freq1)

fwrite(freq,"Rewarewa_freq_untransposed.csv")

vcf_freq<-as.data.frame((t(freq))) %>%

tibble::rownames_to_column() %>%

dplyr::rename(pop = rowname)

vcf_freq<-as.data.frame(vcf_freq)

fwrite(vcf_freq, "Rewarewa_freq_noMiss.csv")

**# DAPC analysis**

library(adegenet)

library(data.table)

library(plotly)

setwd("/Rewarewa/Analyses/")

vcf_freq <- fread( "Rewarewa_freq_noMiss.csv", header=TRUE)

vcf_freq2<-as.data.frame(vcf_freq)

row.names(vcf_freq2)<-paste(vcf_freq2$pop,row.names(vcf_freq2),sep="_")

vcf_freq2$pop<-NULL

grp<-find.clusters(vcf_freq2,max.n=30, n.pca=35, scale=FALSE,

choose.n.clust=FALSE,criterion="min" )

plot(grp$Kstat,type="b")

dapc<-dapc(vcf_freq2, grp = grp$grp, n.pca = 100, n.da = 100,

scale = FALSE, var.contrib = FALSE)

dapc_optim<-optim.a.score(dapc)

dapc<-dapc(vcf_freq2, grp = grp$grp,n.da=100,n.pca=dapc_optim$best)

dapc_coordinates<-dapc$ind.coord

custom_text<-paste0("Population :", pca$names)

cols <- c("deeppink3","darkorange3","steelblue3","aquamarine2")

p <- plot_ly(dapc_coordinates, type = "scatter3d",x = ~LD1 , y = ~LD2, z= ~LD3, text = custom_text, mode = "markers", color = as.factor(pca$clusters), colors = c("deeppink3","darkorange3","steelblue3","aquamarine2"), marker = list(size = 7),width=800,height=600)

p

**# NPStats**

module load samtools

module load bcftools

module unload R/3.3.0

module load R/3.4.3

module load NPStat

cd /Rewarewa/Analyses/bam_files/

for i in *.bam; \

do \

bsub -o/$i.out -e /$i.err -n 1 -J sort$i \

"samtools sort -o /$i.sorted.bam $i"; \

done

ls *.bam.sorted.bam

for BAM in $(ls *.bam.sorted.bam); \

do \

bsub -o /${BAM}.out -e /${BAM}.err -n 1 -J $BAM \

"samtools mpileup -A -f /bam_files/Reference_Genome/FLYE_genome.fasta \

${BAM} > /${BAM}.mpileup"; \

done

for FILE in $(ls *.bam.sorted.bam.mpileup.gz)

do

echo "npstat -n 30 -l 10000 -mincov 25 -maxcov 500 /${FILE}"

done

**# Calculate NPStats Weighted Medians**

library(data.table)

library(spatstat)

setwd("/Rewarewa/Analyses/")

data<-fread("/bam_files/NPStats/WPi_Flye.bam.sorted.bam.mpileup.gz.stats", header = TRUE)

data<-as.data.frame(data)

data<-data[-c(11:18)]

data<-na.omit(data)

weighted.median(data$S, data$length)

weighted.median(data$Watterson, data$length)

weighted.median(data$Pi, data$length)

weighted.median(data$Tajima_D, data$length)

weighted.median(data$var_S, data$length)

weighted.median(data$var_Watterson, data$length)

**# Estimate pairwise Fst**

module unload perl/5.26.0

module load popoolation2

module load samtools

module load bcftools

module unload R/3.3.0

module load R/3.4.3

cd /Rewarewa/Analyses/bam_files/

GENOME="/bam_files/Reference_Genome/FLYE_genome.fasta"

samtools mpileup -A -f $GENOME --bam-list bam.list -o pops2.mpileup

java -ea -Xmx80g -jar /popoolation2-1201/mpileup2sync.jar \

--input pops2.mpileup --output pops2.sync --fastq-type sanger --min-qual 20 --threads 8

library(poolfstat)

setwd("/Rewarewa/Analyses")

pops<-popsync2pooldata(sync.file = "/bam_files/pops2.sync",

poolsizes = c(rep(“30”,35), poolnames = c("BI","BP","CR","DU","GB","GS","HH","K","LB","mb","MG","MP","MS","NP","om","OP","PB","PI","PN","pp","PU","RMim","ROka","RPar","RP","rt","Rwai","T","TR","TS","WA","WE","WN","WP","WPi"),

min.rc = 1, min.cov.per.pool = 50, max.cov.per.pool = 1e+06,

min.maf = 0.05, noindel = TRUE, nlines.per.readblock = 1e+06)

pooldata2genobaypass(pooldata=pops,writing.dir=getwd())

fstpairs<-computePairwiseFSTmatrix(pops,

method = "Anova",

output.snp.values = FALSE)

fstpairs<-as.matrix(fstpairs$PairwiseFSTmatrix)

fstpairs

write.csv(fstpairs, "/PoolFstat_matrix.csv")

**#Estimate IBD**

library(adegenet)

library(ade4)

library(dichromat)

library(geosphere)

library(MASS)

setwd("/Rewarewa/Analyses/")

Dgen<-dist(read.table("/PoolFstat_IBD_matrix.csv",

sep=","))

Dgeo<-read.table("/Rewarewa_lats_longs.csv",header=TRUE,sep=",")

Dgeo<-distm(Dgeo,fun=distVincentyEllipsoid)

ibd<-mantel.randtest(Dgen, Dgeo)

ibd

**# TreeMix Analysis**

module load samtools

module load bcftools

module load vcftools

module load conda

module load R

cd /Rewarewa/Analyses

VCF=/Rewarewa_merged_filtered_maf.vcf.gz

bcftools view \

--min-alleles 2 --max-alleles 2 $VCF | bcftools annotate \

-x INFO,^FORMAT/AD |\

grep -v '^##' |\

cut -f 1-9 --complement |\

sed 's/rewa_flye_rx4_med_srx1_Salsa_aln.counts_GATC.//g' \

> /Treemix.txt

gzip -f Treemix.txt

conda activate /Treemix

for m in {1..30}

do

for i in {1..5}

do

treemix \

-i /Treemix.txt.gz \

-o /TreeMix/Rewarewa.$i.$m.Treemix.stem \

-m $m \

-bootstrap \

-root 9:Rewa_Pool \

-k 1000

done

done

**# Use OptM to determine best number of migration events (M)**

library(OptM)

setwd("/Rewarewa/Analyses/TreeMix/")

folder <- "/Rewarewa/Analyses/TreeMix/"

optM_evanno = optM(folder, method = "Evanno", tsv = 'Rewarewa_Treemix_evanno.tsv',thresh = 0.05)

**# Plot TreeMix results**

Rscript -e "source('/TreeMix/treemix-1.13/src/plotting_funcs.R');\

png('Rewarewa.3.1.Treemix.stem.png',width=900,height=800);\

plot_tree('Rewarewa.3.1.Treemix.stem', cex=1,lwd=2, arrow = 0.3, plotnames = F);\

dev.off()" 2>&1 >/dev/null

Rscript -e "source('/TreeMix/treemix-1.13/src/plotting_funcs.R');\ png('/TreeMix1/Rewarewa.1.Treemix.residuals.png',width=900,height=800);\ plot_resid('/TreeMix1/Rewarewa.3.1.Treemix.stem','/pop.order');\

dev.off()" 2>&1 >/dev/null

**# Demographic Modeling**

module load samtools

module load bcftools

module load vcftools

workDir= /Rewarewa/Analyses/

cd $workDir

vcftools --gzvcf Rewarewa_merged_filtered2.vcf.gz --keep WestCoas_pops --recode --out WestCoas_MAF00

bcftools query -f '%CHROM %POS %REF %ALT [ %AD]\n' $workDir/WestCoas_MAF00.recode.vcf \

> $workDir/WestCoas_MAF00.txt

library(tidyr)

library(data.table)

library(dplyr)

library(stringr)

setwd("/Rewarewa/Analyses")

pool<-read.table("WestCoas_MAF00.txt")

colnames(pool)<-c("CHROM","POS","REF","ALT","2:Rewa_Pool","5:Rewa_Pool","10:Rewa_Pool","11:Rewa_Pool","15:Rewa_Pool","16:Rewa_Pool","19:Rewa_Pool","21:Rewa_Pool","22:Rewa_Pool","30:Rewa_Pool","32:Rewa_Pool")

pool$LOCI<-paste(pool$CHROM,pool$POS,pool$REF,pool$ALT,sep=":")

pool$CHROM<-NULL

pool$POS<-NULL

pool$REF<-NULL

pool$ALT<-NULL

pool_0<- melt(pool,"LOCI")

pool<-pool_0 %>%

separate(value, c("REF", "ALT"), ",")

pool$REF<-as.numeric(pool$REF)

pool$ALT<-as.numeric(pool$ALT)

pool$DP<-(pool$REF+pool$ALT)

pool$AAF<-(pool$ALT/pool$DP)

pool$RAF<-(pool$REF/pool$DP)

pool_REF<-data.frame(pool$LOCI,pool$variable,pool$RAF)

pool_ALT<-data.frame(pool$LOCI,pool$variable,pool$AAF)

poolREF<-dcast(pool_REF, pool.LOCI ~ pool.variable)

poolALT<-dcast(pool_ALT, pool.LOCI ~ pool.variable)

loci<-poolREF$pool.LOCI

loci<-data.frame(loci)

poolREF$pool.LOCI<-NULL

poolALT$pool.LOCI<-NULL

poolREF$RAF_av<-rowMeans(poolREF)/rowMeans(!!poolREF)

poolALT$AAF_av<-rowMeans(poolALT)/rowMeans(!!poolALT)

poolREF[poolREF == "NaN"] <- 0.000

poolALT[poolALT == "NaN"] <- 0.000

pool<-data.frame(loci$loci,poolREF$RAF_av,poolALT$AAF_av)

fwrite(pool, "WestCoast_averages.csv")

vcf_ex<-read.csv("WestCoast_averages.csv")

vcf_ex<-vcf_ex %>%

separate(loci.loci, c("CHROM","POS","REF", "ALT"), ":")

colnames(vcf_ex)<-c("CHROM","POS","REF","ALT","RAF","AAF")

vcf_ex$ID<-paste(vcf_ex$CHROM,vcf_ex$POS,sep=":")

vcf_ex$x_alt<-(vcf_ex$AAF*30)

vcf_ex$x_ref<-(30-vcf_ex$x_alt)

data<-na.omit(vcf_ex)

data$folded<- '1'

data$x<-ifelse(data$folded == 0,data$x_alt,data$x_ref)

data$x<-floor(data$x)

data$n<-(data$x_ref+data$x_alt)

sfs<-select(data,"POS","x","n","folded")

colnames(sfs)<-c("position","x","n","folded")

sfs$x<-floor(sfs$x)

fwrite(sfs,"SFS_WestCoast.txt",sep="\t")

joint<-select(data,"ID","x_alt")

joint$x_alt<-floor(joint$x_alt)

fwrite(joint,"joint_WestCoast.txt",sep="\t")

pop1<-read.table("joint_WestCoast.txt",header=TRUE)

pop2<-read.table("joint_SouthIsland.txt",header=TRUE)

pop1<-as.data.frame(pop1)

pop2<-as.data.frame(pop2)

data<- merge(pop1,pop2,by="ID")

data<-na.omit(data)

df<-select(data,"x_alt.x","x_alt.y")

input_names = names(df)

counts<- df %>% count_(input_names) %>% unite_("ComboVar",input_names,sep=",")

counts<-counts %>%

separate(ComboVar, c("x", "y"), ",")

counts<-as.data.table(counts)

m <- as.matrix(dcast.data.table(data=counts, x ~ y, value.var="n", fill=0)[,-1, with=FALSE])

rownames(m)<-colnames(m)

m<-m[order(as.numeric(rownames(m))),order(as.numeric(colnames(m)))]

write.matrix(m,"WestCoast_SouthIsland_matrix.txt",sep="\t")

module load conda

module load R

conda activate sweepfinder2

cd /Rewarewa/Analyses

SweepFinder2 -f SFS_WestCoast.txt SFS_WestCoast_out

DIR="/fsc26_linux64/"

PREFIX="WestCoast_neutral" ##as example

WORKDIR="/Rewarewa/Analyses/Demo_models/${PREFIX}"

for i in {1..100}

do

mkdir -p run$i

cp ${PREFIX}.tpl ${PREFIX}.est ${PREFIX}_DAFpop0.obs run$i"/"

cd run$i

$DIR/fsc26 -t $PREFIX.tpl -n 100000 -d -e $PREFIX.est -0 -M -L 40 -q -C10

cd ..

done

**# Estimate best run**

## fsc-selectbestrun.sh bash script by Joana Meier

m=-1000000000000000

p=$1

c=0

best="xxxx"

diff=0

diffBest=0

RUN='ls -d /powerplant/workspace/hrpemk/Rewarewa/Analyses/Demo_models/${PREFIX}/run*'

for i in $RUN;

do

a=$(ls $i | grep '.tpl' | sed s'/.tpl//')

# if the file is in a subdirectory

if [ -e $i/$a/$a.bestlhoods ]

then

l=$(cat $i/$a/$a.bestlhoods | awk '{print $(NF-1)}' | tail -1 | cut -f 1 -d ".")

diff=$(cat $i/$a/$a.bestlhoods | awk '{print $NF-$(NF-1)}' | tail -1 | cut -f 1 -d ".")

((c++))

else

echo "no .bestlhoods file found in "$i

fi

if [ $l -gt $m ]

then

m=$l; x=$i; best=$i;

diffBest=$diff

fi

done

if [ -z ${x+1} ]

then echo "Error: No run with lik>-1000000000000000"

else mkdir bestrun

cp $x/* ./bestrun/

cp $x/$a/* ./bestrun/

fi

echo -e "\n"$c" bestlhoods files found, "$best" with "$diffBest" Lhood diff fits best."

cd $WORKDIR/bestrun

Rscript -e 'bestlhoods<-read.delim(paste("WestCoast_neutral.bestlhoods",sep=""));

est<-readLines(paste("WestCoast_neutral.est",sep=""));

k<-(grep("RULES",est))-(grep("//all Ns are",est)+1);

AIC<-2*k-2*(bestlhoods$MaxEstLhood/log10(exp(1)));

deltaL<-bestlhoods$MaxObsLhood-bestlhoods$MaxEstLhood;

write.table(cbind(deltaL,AIC),paste("WestCoast_neutral",sep=""),row.names = F,col.names = T,sep = "\t",quote = F)'

cat run{1..100}/${PREFIX}/${PREFIX}.bestlhoods | grep -v MaxObsLhood | sort -k 2 > ${PREFIX}_100runs.txt

WORKDIR="/Rewarewa/Analyses/Demo_models/${PREFIX}/bestrun/Final_Run"

for i in {1..100}

do

mkdir -p run$i

cp ${PREFIX}.tpl ${PREFIX}.est ${PREFIX}_DAFpop0.obs run$i"/"

cd run$i

$DIR/fsc26 -t $PREFIX.tpl -n 100000 -d -e $PREFIX.est -0 -M -L 40 -q -C10

cd ..

done

m=-1000000000000000

p=$1

c=0

best="xxxx"

diff=0

diffBest=0

RUN='ls -d /powerplant/workspace/hrpemk/Rewarewa/Analyses/Demo_models/${PREFIX}/bestrun/Final_run/run*'

for i in $RUN;

do

a=$(ls $i | grep '.tpl' | sed s'/.tpl//')

# if the file is in a subdirectory

if [ -e $i/$a/$a.bestlhoods ]

then

l=$(cat $i/$a/$a.bestlhoods | awk '{print $(NF-1)}' | tail -1 | cut -f 1 -d ".")

diff=$(cat $i/$a/$a.bestlhoods | awk '{print $NF-$(NF-1)}' | tail -1 | cut -f 1 -d ".")

((c++))

else

echo "no .bestlhoods file found in "$i

fi

if [ $l -gt $m ]

then

m=$l; x=$i; best=$i;

diffBest=$diff

fi

done

if [ -z ${x+1} ]

then echo "Error: No run with lik>-1000000000000000"

else mkdir bestrun

cp $x/* ./bestrun/

cp $x/$a/* ./bestrun/

fi

echo -e "\n"$c" bestlhoods files found, "$best" with "$diffBest" Lhood diff fits best."

cd $WORKDIR/bestrun/Final_run

Rscript -e 'bestlhoods<-read.delim(paste("WestCoast_neutral.bestlhoods",sep=""));

est<-readLines(paste("WestCoast_neutral.est",sep=""));

k<-(grep("RULES",est))-(grep("//all Ns are",est)+1);

AIC<-2*k-2*(bestlhoods$MaxEstLhood/log10(exp(1)));

deltaL<-bestlhoods$MaxObsLhood-bestlhoods$MaxEstLhood;

write.table(cbind(deltaL,AIC),paste("WestCoast_neutral",sep=""),row.names = F,col.names = T,sep = "\t",quote = F)'

cat run{1..100}/${PREFIX}/${PREFIX}.bestlhoods | grep -v MaxObsLhood | sort -k 2 > ${PREFIX}_100runs.txt

**# GEA Analyses**

#LFMM

library(vcfR)

library(data.table)

library(lfmm)

library(qvalue)

library(ggplot2)

library(lattice)

library(tidyverse)

setwd("/Rewarewa/Analyses")

vcf <- read.vcfR( file = "/Rewarewa/Analyses/Rewarewa_merged_filtered.vcf.gz", verbose = FALSE )

ad<-extract.gt(vcf, element = "AD")

freq1<-AD_frequency(ad, delim = ",", allele = 2L)

freq1<-as.data.frame(freq1, rownames=rownames)

miss<-freq1[!complete.cases(freq1),]

freq <- na.omit(freq1)

fwrite(freq,"Rewarewa_lfmm_untransposed.csv")

vcf_freq<-as.data.frame((t(freq))) %>%

tibble::rownames_to_column() %>%

dplyr::rename(pop = rowname)

vcf_freq<-as.data.frame(vcf_freq)

str(vcf_freq[1:10])

fwrite(vcf_freq, "Rewarewa_lfmm_noMiss.csv")

Y <- fread("/Rewarewa/Analyses/Rewarewa_lfmm_noMiss.csv", header = TRUE)

Y$pop <- NULL

names(Y) <- NULL

**# Altitude (as example)**

X<-fread("Rewarewa_lfmm_covariate.std",header =FALSE)

X1 <- X$V1

mod.lfmm <- lfmm_ridge(Y = Y, X = X1, K = 4, algorithm = "alternated", it.max = 100)

pv <- lfmm_test(Y = Y, X = X1, lfmm = mod.lfmm, calibrate = "gif")

df<-as.data.frame(pv)

write.csv(df,"Altitude_lfmm.csv")

pvalues <- pv$calibrated.pvalue

pvs<-as.data.frame(pvalues)

write.csv(pvs,"Altitude_pvalues.csv")

x<-fread("Altitude_pvalues.csv",header =TRUE)

colnames(x)<- c("ID", "pvalue")

qqplot(rexp(length(x$pvalue), rate = log(10)),

-log10(x$pvalue), xlab = "Expected quantile",

pch = 19, cex = .4)

abline(0,1)

plot(-log10(x$pvalue),

pch = 19,

cex = .2,

xlab = "SNP", ylab = "-Log P",

col = "grey")

abline(h = 1.3,

col = "red")

loci<-fread("/Rewarewa/Analyses/Rewarewa_lfmm_noMiss_loci_transposed.txt",header=TRUE)

pvalues.loci<-cbind(loci,x)

colnames(pvalues.loci)<-c("CHROM_POS","ID","p.values")

hist(pvalues.loci$p.values, col = "lightblue")

p.values<-as.data.frame(pvalues.loci$p.values)

colnames(p.values)<-"pvalues"

qobj <- qvalue(p.values$pvalues)

summary(qobj)

q.values<-qobj$qvalues

lfdr<-qobj$lfdr

q.values<-as.data.frame(q.values)

lfdr<-as.data.frame(lfdr)

p.values$qvalue<-q.values$q.values

p.values$lfdr<-lfdr$lfdr

p.values$CHROM<-pvalues.loci$CHROM

p.values$POS<-pvalues.loci$POS

alpha <- 0.1

outliers <- which(p.values$qvalue < alpha )

length(outliers)

filtered<-p.values %>% filter(qvalue < 0.1)

str(filtered)

fwrite(filtered,"Rewarewa_lfmm_qvalues_Altitude.txt",sep="\t")

**#RDA Analysis**

library(vegan)

library(data.table)

library(qvalue)

library(psych)

library(tidyverse)

setwd("//Rewarewa/Analyses/RDA")

gen <- fread("/Rewarewa/Analyses/Rewarewa_lfmm_noMiss.csv", header = TRUE)

env <- fread("/Rewarewa/Analyses/LFMM2/Rewarewa_lfmm_covariate.std",header =FALSE)

meta <- fread("/Rewarewa/Analyses/Rewarewa_metadata.csv")

colnames(env) <- c("Altitude","SoilPH","SoilCarbon","Slope","SoilSize","Bio1","Bio2","Bio3","Bio12","Bio15")

rda <- rda(gen ~ ., data=env, scale=T)

rda

RsquareAdj(rda)

summary(eigenvals(rda, model = "constrained"))

levels(meta$Gene_pool) <- c("South_Island","Northland","East_Coast","West_Coast")

cluster <- meta$Gene_pool

colvec <- c("deeppink3","darkorange3","steelblue3","aquamarine2")

plot(rda, type="n", scaling=3)

points(rda, display="species", pch=20, cex=0.7, col="gray32", scaling=3)

points(rda, display="sites", pch=20, cex=1.3, col = colvec, scaling=3, bg=bg[meta$Gene_pool])

text(rda, scaling=3, display="bp", col="#0868ac", cex=1)

legend("bottomright", legend=levels(cluster), bty="n", col="gray32", pch=21, cex=1, pt.bg=bg)

plot(rda, type="n", scaling=3)

points(rda, display="species", pch=20, cex=0.7, col="gray32", scaling=3, choices=c(1,3))

points(rda, display="sites", pch=20, cex=1.3, col=colvec, scaling=3, bg=bg[meta$Gene_pool], choices=c(1,3))

text(rda, scaling=3, display="bp", col="#0868ac", cex=1, choices=c(1,3))

legend("bottomright", legend=levels(meta$Gene_pool), bty="n", col="gray32", pch=21, cex=1, pt.bg=bg)

load.rda <- summary(rda)$species[,1:3]

hist(load.rda[,1], main="Loadings on RDA1")

hist(load.rda[,2], main="Loadings on RDA2")

hist(load.rda[,3], main="Loadings on RDA3")

outliers <- function(x,z){

lims <- mean(x) + c(-1, 1) * z * sd(x) # find loadings +/-z sd from mean loading

x[x < lims[1] | x > lims[2]] # locus names in these tails

}

cand1 <- outliers(load.rda[,1],3)

cand2 <- outliers(load.rda[,2],3)

cand3 <- outliers(load.rda[,3],3)

length(cand1)

length(cand2)

length(cand3)

ncand <- length(cand1) + length(cand2) + length(cand3)

ncand

cand1 <- cbind.data.frame(rep(1,times=length(cand1)), names(cand1), unname(cand1))

cand2 <- cbind.data.frame(rep(2,times=length(cand2)), names(cand2), unname(cand2))

cand3 <- cbind.data.frame(rep(3,times=length(cand3)), names(cand3), unname(cand3))

colnames(cand1) <- colnames(cand2) <- colnames(cand3) <- c("axis","snp","loading")

cand <- rbind(cand1, cand2, cand3)

cand$snp <- as.character(cand$snp)

foo <- matrix(nrow=(ncand), ncol=10) # 10 columns for 10 predictors

colnames(foo) <- c("Altitude","SoilPH","SoilCarbon","Slope","SoilSize","Bio1","Bio2","Bio3","Bio12","Bio15")

for (i in 1:length(cand$snp)) {

nam <- cand[i,2]

snp.gen <- gen[,..nam]

foo[i,] <- apply(env,2,function(x) cor(x,snp.gen))

}

cand <- cbind.data.frame(cand,foo)

head(cand)

length(cand$snp[duplicated(cand$snp)])

cand <- cand[!duplicated(cand$snp),]

for (i in 1:length(cand$snp)) {

bar <- cand[i,]

cand[i,14] <- names(which.max(abs(bar[4:13]))) # gives the variable

cand[i,15] <- max(abs(bar[4:13])) # gives the correlation

}

colnames(cand)[14] <- "predictor"

colnames(cand)[15] <- "correlation"

table(cand$predictor)

fwrite(cand,"Rewarewa_RDA_sig_SNPs.txt")

Altitude <- cand %>% filter(predictor == "Altitude")

Altitude[,3:13] <- NULL

head(Altitude)

fwrite(Altitude,"Rewarewa_RDA_altitude.txt")

**# Compare LFMM and RDA results**

df_lfmm <- fread("/Rewarewa/Analyses/LFMM2/Rewarewa_lfmm_qvalues_Altitude.txt")

df_RDA <- fread("/Rewarewa/Analyses/RDA/Rewarewa_RDA_altitude_25SD.txt")

df_lfmm$snp <- df_lfmm$CHROM

df <- intersect(df_lfmm$snp, df_RDA$snp)

altitude <- as.data.frame(df)

altitude$variable <- 'Altitude'

altitude

fwrite(df,"Rewarewa_significant_SNPs_LFMM_RDA.txt")

**Supplementary Scripts 3: Annotation**

**# Install braker3**

*# Download GeneMark-ES/ET/EP and the license key, install according to instructions by GeneMark authors (e.g. you have to unpack the archive and the key, and move the license key)*

*# Install or activate Singuarlity (on most HPC it's "module load singularity" if available)*

singularity build braker.sif docker://katharinahoff/braker-notebook:devel

singularity exec braker.sif [braker.pl](http://braker.pl/)

**# Mask Repeats**

#Download repeatmodeler using conda

BuildDatabase -name rewarewa_repeatmodelerDB FLYE_genome.fasta

RepeatModeler -database Rewa -pa 8 -LTRStruct >& run.out &

RepeatMasker -pa 8 -gff -nolow -lib consensi.fa.classified ./RM_40548.MonNov211322012022/round-6/sampleDB-6.fa

**# Quality Contro**l

Install fastqc using bioconda

Run fastqc

./fastqc R1.fastq.gz –outdir /path/

**# Data cleaning using trim_galore**

trim_galore --paired --illumina -j 2 R1.fastq.gz R2.fastq.gz

#Remove 1-50bp from each read.

trim_galore --paired --hardtrim3 100 R1.fastq.gz R2.fastq.gz

**# Index genome**

mkdir rewa_index

STAR --runThreadN 6 --runMode genomeGenerate --genomeDir rewa_index --genomeFastaFiles FLYE_genome.fasta --genomeSAindexNbases 13

#Note: --genomeSAindexNbases 13 was implemented as the default 14 was too large for the genome size and would lead to a seg-fault at mapping step.

**# Map your reads**

/public/groups/cgl/local/bin/STAR --runThreadN 8 --genomeDir ./GenomeDir --outFileNamePrefix Rewa_STARalignments --readFilesIn R1.fastq R2.fastq

**# Convert Sam to Bam**

samtools view --threads 8 -b -o Rewa_STARalignmentsAligned.bam Rewa_STARalignmentsAligned.out.sam >& run.out &

**# Sort Bam**

samtools sort -m 7G -o Rewa_STARalignmentsAligned_sorted.bam -T rewa_temp --threads 8 Rewa_STARalignmentsAligned.bam >& run.out &

**# Generate Alignment Stats**

picard CollectAlignmentSummaryMetrics REFERENCE_SEQUENCE=FLYE_genome_header.fasta INPUT=Rewa_STARalignmentsAligned_sorted.bam OUTPUT=Rewa_STARalignmentsAligned.stats

**# Run BRAKER3**

singularity shell -B /BRAKER/

braker.sif braker.pl --species=Rewarewa --genome=FLYE_genome_header.fasta.masked --bam=Rewa_STARalignmentsAligned_sorted.bam >& test_real.log &

**# Run BUSCO**

/public/home/anmmccar/.conda/envs/BUSCO/bin/busco --in euk_coding.fa --out euk_busco -m tran
